# Resolving acne with optimized adapalene microspongeal gel, in vivo and clinical evaluations

**DOI:** 10.1038/s41598-024-51392-1

**Published:** 2024-01-16

**Authors:** Rania M. Yehia, Mahmoud H. Teaima, Maha H. Ragaie, Mohamed M. Elmazar, Dalia A. Attia, Mohamed A. El-Nabarawi

**Affiliations:** 1 Department of Pharmaceutics and Pharmaceutical Technology, Faculty of Pharmacy, The British University in Egypt (BUE), Suez Desert Road, El Sherouk City, Cairo, 1183 Egypt; 2 Department of Pharmaceutics and Industrial Pharmacy, Faculty of Pharmacy, Cairo University, Cairo, Egypt; 3 Department of Dermatology, STDs and Andrology, Faculty of Medicine, Minia University, Al Minya, Egypt; 4 Department of Pharmacology and Toxicology, Faculty of Pharmacy, The British University in Egypt (BUE), Cairo, Egypt

**Keywords:** Translational research, Drug delivery

## Abstract

In our pursuit of enhancing acne treatment while minimizing side effects, we developed tailored Adapalene microsponges (MS) optimized using a Box–Behnken design 3^3^. The independent variables, Eudragit RS100 percentage in the polymer mixture, organic phase volume, and drug to polymer percentage, were explored. The optimized formulation exhibited remarkable characteristics, with a 98.3% ± 1.6 production yield, 97.3% ± 1.64 entrapment efficiency, and a particle size of 31.8 ± 1.1 µm. Notably, it achieved a 24 h cumulative drug release of 75.1% ± 1.4. To delve deeper into its efficacy, we evaluated the optimized microspongeal-gel in vitro, in vivo, and clinically. It demonstrated impressive retention in the pilosebaceous unit, a target for acne treatment. Comparative studies between our optimized Adapalene microspongeal gel and marketed Adapalene revealed superior performance. In vivo studies on Propionibacterium acnes-infected mice ears showed a remarkable 97% reduction in ear thickness, accompanied by a significant decrease in inflammatory signs and NF-κB levels, as confirmed by histopathological and histochemical examination. Moreover, in preliminary clinical evaluation, it demonstrated outstanding effectiveness in reducing comedonal lesions while causing fewer irritations. This not only indicates its potential for clinical application but also underscores its ability to enhance patient satisfaction, paving the way for future commercialization.

## Introduction

Acne vulgaris is one of the top ten prevalent diseases, affecting 80–90% of teenagers^1^. Despite its commonality, acne significantly affects the quality of life and can lead to mental health issues, potentially resulting in depression and suicidal thoughts^2^. Thus, the competent treatment of acne is essential for refined psychosocial impact^3^.

Acne treatment involves both oral and topical therapies. Hitherto, oral therapy is reserved for severe cases^4^. Topical therapy comprises keratolytics and antibiotics, in addition to retinoids, which are considered a first line of treatment for mild to moderate acne^5^. One of the most frequently prescribed topical retinoids is adapalene (ADA)^6^. Adapalene was first approved by the FDA in 1996 for acne treatment in patients 12 years of age or older and as an over-the-counter (adapalene 0.1% gel) acne treatment in 2016^7^. Even yet, it has severe local side effects, including skin irritation, desquamation, burning, stinging, and increased photosensitivity, which may eventually make patients less compliant^8^. Studies have reported that due to the associated side effects, patients usually discontinue treatment before experiencing its effects^9,10^.

Consequently, carrier systems have been proposed as a solution to optimize the usage of ADA in the treatment of acne. Various types of carriers for loading ADA alone or in combination with other antiacne agents have been investigated, including solid lipid microparticles, transferosomes, noisomes, liposomes, microspheres and microemulsion^11^. However, while microsponges are widely advocated for topical delivery due to their numerous advantages, there is a notable absence of prior in vivo or clinical studies investigating their applicability specifically for the topical delivery of ADA^11,12^.

Microsponges (MS) drug delivery system is a polymeric system consisting of porous microparticles, each being spherical with interconnected pores (size: 5–300 µm) resembling a noncollapsible sponge structure^12^. MS has certain qualities that single out them as a favorable carrier system for topical delivery. These qualities include their competence to entrap large quantities of active ingredients of different natures within its matrix or on its surface. This enables the controlled delivery of the drug, with minimized drug exposure to the skin surface abating the dermal irritation effect. The size of MS prevents deep skin permeation, averting systemic absorption of the entrapped drug^13^. MS exhibit high stability to pH (1–11) and temperature variations (up to 130 °C), and their minute pore size (< 0.25 μm) provides immunity against bacterial infiltration^14,15^. Both the liquid‒liquid suspension polymerization method and quasi-emulsion solvent diffusion method are simple methods for MS preparation^15^. MS are thereby considered stable, cost effective and prone to heightened patient compliance^12,13,15^. However, optimizing the formula for ADA-loaded MS involves multiple variables, necessitating the use of software for practical optimization over traditional trial and error methods^16^.

We hypothesized that the encapsulation of ADA in MS would augment combating acne disease in conjunction with curtailing both the side effects and the systemic absorption of ADA. Therefore, the ruling objective was to design, characterize and optimize MS loaded with ADA using the Box‒Behnken design (3^3^ factorial) to generate an optimized ADA-MS formula to be incorporated into the hydrogel base. Evaluate the proposed formula ex vivo for ADA distribution into skin layers both quantitively and qualitatively. Furthermore, we investigated the in vivo antiacne efficacy using a *Propionibacterium acnes*-induced acne mice model, accompanied by histopathological and histochemical studies. Finally, a split face double-blinded preliminary clinical study was conducted.

## Materials and methods

### Materials

Adapalene (ADA) was kindly donated by Borg Pharmaceutical Industries (Alexandria, Egypt). Adapalene gel, 0.1% ADA, batch number 0111195, Borg Pharmaceutical Industries (Alexandria, Egypt) was bought from the neighborhood pharmacy. Evonik Operations GmbH (Essen, Germany) donated Eudragit RS 100 (EUD). Egyptian International Pharmaceutical Industries Co., EIPICO, 10th of Ramadan City, Egypt, generously donated ethyl cellulose (EC) and polyvinyl alcohol (PVA). From El-Nasr Pharmaceutical Chemicals Co. in Cairo, Egypt, we bought dichloromethane, disodium hydrogen phosphate, potassium dihydrogen phosphate, Carbopol 934 propylene glycol, and triethanolamine. Cellulose membrane dialysis tubing with a molecular weight cutoff of 12,000 Dalton was purchased from Sigma‒Aldrich Co. (St Louis, MO, USA). Tetrahydrofuran (THF), ethanol, and Dil fluorescent dye were acquired from Fisher Scientific in Massachusetts, USA. We prepared all aqueous solutions using distilled water generated in-house (Aquatron Water Still, A4000D, UK).

### Methods

#### Development of experimental design

This investigation was optimized using Box‒Behnken design (BBD), a 3-factor, 3-level (3^3^) experimental design (Design-Expert v.13.0.0, Stat-Ease, Inc., Minneapolis, MN, USA). There was a total of 15 runs produced, of which 12 represented the midpoints of each edge of the multidimensional cube and the remaining 3 were replicas of the cube's center. Three independent variables—Eudragit RS100 percent in polymer mixture (A), organic phase volume (B), and drug to polymer mixture percent (C)—were chosen at three levels in Table 1 based on the findings of a prior screening research^17^. Production yield% (Y1), entrapment efficiency% (Y2), particle size% (Y3), and cumulative drug release after 24 h% (Y4) were the dependent variables that were examined for the independent factors' effects on them. Table 2 displays the prepared ADA-MS components in accordance with BBD.

**Table 1:** Levels of independent and dependent variables by Box–Behnken design for ADA-MS.

Investigated variables	Levels		
	− 1	0	1
Eudragit RS100 percent in polymer mixture (%) (A)	0	50	100
Organic phase volume (ml) (B)	15	20	25
Drug to polymer mixture percent (%) (C)	70	75	80

Dependent variables	Constrains		
Production yield (%) (Y1)	Maximize		
Entrapment efficiency (%) (Y2)	Maximize		
Particle size (µm) (Y3)	In range		
Cumulative drug release after 24 h (%) (Y4)	Maximize		

#### Preparation of ADA-loaded microsponges

The quasi-emulsion solvent diffusion method was used to produce microsponges formulae based on the values in Table 2^18^. The internal organic phase was prepared by dispersing the appropriate amounts of ADA and polymer(s) in dichloromethane (DCM). Knowing this, either pure Ethyl cellulose (EUD 0%), a 1:1 ratio of EC and EUD, or pure Eudragit RS100 (EUD 100%) made up the polymer mixture. The organic phase was then subjected to a 10-min sonication process in a bath sonicator (Germany's Elmasonic S60H). The external aqueous media was formed using 100 mL of distilled water and 0.1% of the emulsifying agent PVA, which was then heated to 70 °C using a magnetic stirrer and hotplate (DAIHAN Scientific, Korea) before being allowed to cool. Subsequently, the organic phase was added to the aqueous phase in portions, and the mixture was continuously stirred for 2 h at room temperature using an overhead stirrer (HS-30D, DAIHAN Scientific, Korea) at 1500 rpm. The produced microsponges were subsequently processed in accordance with prior research^17^.

#### Characterization of the prepared ADA-MS formulae

The produced Adapalene loaded microsponges (15 formulae) were examined for production yield % (Y1), entrapment efficiency % (Y2), particle size (Y3), and cumulative drug release after 24 h % (Y4). Furthermore, the morphological features of all the prepared formulae were evaluated.

##### Production yield % (P.Y.%) (Y1)

Percentage P.Y. was measured via obtaining the original amounts of added materials together with the recovered amount of MS and using the following formula to calculate it^19^.

$$Production\; Yield \left( \% \right) = \frac{Practical \;mass \;of\; microsponges}{{Theoretical \;mass \left( {polymer + drug} \right)}} \times 100$$

##### Entrapment efficiency (EE%) (Y2)

Entrapment efficiency was estimated by first breaking down a preset amount of the retrieved ADA-MS in tetrahydrofuran according to the method mentioned in a preceding research study^17^. The succeeding equation^20^ was used to estimate the percentage of drug entrapped after the experiment was carried out three times for each sample.

$$EE \left( \% \right) = \frac{Actual\; amount\; of\; drug\; in\; the\; formulation}{{Theoretical\; amount \;of \;drug\; in \;the\; formulation}} \times 100$$

##### Particle size (Y3)

The particle size was determined using a Malvern Mastersizer (Malvern Mastersizer 3000, Malvern, UK). Before measuring the sample in the instrument, ADA-MS were dispersed in double-distilled water to make sure the light scattering signal (given by the particles count per second) was within the equipment's sensitivity range. All measurements were made in triplicate, and the analysis was performed at 25 °C and an angle of detection of 90°. The degree of consistency d (0.9) μm unit was used to express the average particle size^21^.

##### Cumulative drug release after 24 h % (Y4)

By employing the dialysis bag method and a release medium made up of a 60:40 v/v mixture of phosphate buffer (pH 5.5) and Tetrahydrofuran (THF) and cellulose acetate membrane (molecular weight cutoff of 12,000 Da), it was possible to evaluate the in vitro release profile of adapalene from ADA-MS. Media (3 ml) containing 5 mg of formula was transferred into a bag with the two ends tied by strings. After that, the bag was submerged into vials holding 30 ml of release and placed in a shaker bath for 24 h at 37 °C and 100 rpm. At each time period, samples (1 ml) were taken from the receptor compartment. Fresh medium was added in lieu of the samples to maintain sink conditions. The samples were examined using the UV spectrophotometric method at a wavelength of 235 nm (V-630, Jasco, Japan). The cumulative drug release after 24 h % (Q24h) was determined^22^. The experiment was repeated three times.

##### Statistical analysis of the investigated parameters

The evaluation of the effects of the independent variables on the dependent variables was displayed by response surface plots, which allowed the determination of the statistical significance of the BBD. ANOVA was then conducted on the experimental data to address the statistical significance of the model. For the regression analysis and the graphical display, the statistical software Design-Expert v.13.0.0 (Stat-Ease, Inc., Minneapolis, MN, USA) was employed. For the model terms, a p value less than 0.05 was regarded as significant. Following data analysis, linear correlations were obtained in terms of coded variables to quantify the response values for each response.

#### Optimum formula selection and evaluation

The optimized formula was deduced using the BBD by applying constraints on all independent variables to obtain high production yield, entrapment efficiency, cumulative drug release after 24 h % and optimum particle size, with a desirability near 1. The suggested optimized formula was then prepared and subjected to further validation and evaluation. In order to check the validity of the calculated optimal formulation factors and predicted responses given by the software, the responses P.Y.%, E.E.%, P.S. and Q24h of the optimized formula were assayed. The predicted values were paralleled with experimental values, and the bias percent was calculated according to the following equation^23^.

$$Bias{ }\left( {\text{\%}} \right) = { }\frac{{Predicted{ }\;value - Observed\;{ }value}}{{Observed{ }\;value}} \times 100$$

SEM (Thermo Scientific FEG SEM Quattro model, USA) was also used to analyze the morphological characteristics of ADA and optimized ADA-MS, where the sample was brushed directly onto the studs. The photos were recorded using a low vacuum detector and a high voltage of 10 kV. Replicate analysis was used to evaluate the morphological reproducibility. Multiple magnifications were used to obtain the images^24^.

#### Preparation of gel containing ADA-OPTMS

Optimized Adapalene microsponges gel (ADA-OPTMS gel) was prepared using Carbopol 934 polymer as the gel base. With constant stirring using a magnetic stirrer, Carbopol 934 was dissolved in double-distilled water (1% w/v) and allowed to soak for 2 h at room temperature. A preservative was added after complete hydration, continuously stirred until complete dissolution, and then allowed to stand for 30 min to release any trapped air. Appropriate weighed quantity of ADA-OPTMS (equivalent to 0.1% ADA w/w) was dispersed in a penetration enhancer mixture of propylene glycol and ethanol (5% w/w each) and then added to the above dispersion. The obtained viscus dispersion was neutralized using triethanolamine till pH 7, yielding a 0.1%w/w Adapalene loaded optimized microspongeal gel. A reference gel (ADA-gel) was also prepared using the same previous procedure, replacing the ADA-OPTMS with 0.1%w/w of pure ADA. Formulations were stored at room temperature^25^ for further characterization.

#### Characterization of the prepared ADA-OPTMS gel

##### Physical examination of the prepared gel

The prepared ADA-OPTMS gel formulation was inspected visually for color and homogeneity^26^. The odor was also checked. Together with the assessment of the gel texture in terms of stickiness and grittiness via mildly rubbing the gel between two fingers^27^.

##### Determination of the pH of the prepared gel

The pH of the prepared ADA-OPTMS gel was determined using a pH meter (JENWAY, Staffordshire, UK). A solution containing 1 g of ADA-OPTMS gel in 50 ml of distilled water was prepared, and the pH was measured^28^. The results obtained were the average of three determinations.

##### Spreadability of the prepared gel

The spreadability of the prepared ADA-OPTMS gel was evaluated by placing 0.5 g gel within a 1 cm diameter premarked circle on a glass plate over which a second glass plate was placed. A weight of 500 g was allowed to rest on the upper glass plate for 5 min, and then the difference between the initial diameter of the gel and the spread diameter was obtained. Spread circle diameters were measured in centimeters and used as benchmarks for spreadability^29^. The outcomes were the average of three assessments.

##### Rheological studies on the prepared gel

The viscosity of the prepared ADA-OPTMS gel was assessed employing an Anton Paar rheometer (Anton Paar MCR, 301; Australia) using a cone and plate viscometer sensor of 2.5 cm diameter, equipped with instrument software Rheoplus. The formulation was placed in the sample holder and maintained at 4 °C. Then, the viscosity (η) (Pa s) and shear stress (τ) were measured as a function of shear rate (γ) in s^−1^, ranging from 0.1 to 100 s^−1^. Plotting the shear rate as a function of shear stress led to the generation of a complete rheogram. The outcomes were an average of three assessments^30^.

##### Determination of drug content in the prepared gel

The ADA content of the prepared ADA-OPTMS gel was measured by sonicating 0.5 g of gel with 50 ml tetrahydrofuran. Then, the 0.45 μm filtered sample was analyzed using a UV spectrophotometer at λ 237 nm^26^. This experiment was repeated three times.

#### Ex vivo skin deposition study for the prepared gels

##### Skin preparation

Mouse skin was chosen for the skin deposition evaluation. Male BALB/c mice weighing 20–25 g were mercifully sacrificed via cervical dislocation. This study protocol was permitted by the research ethics committee for experimental and clinical studies at the Faculty of Pharmacy, Cairo University-Cairo-Egypt (no. PI-2287). After hair removal, the dorsal abdominal skins were depilatory removed. Using tweezers, the subcutaneous fat was separated, and the skin fragments were then cleaned with buffer, checked for integrity, and kept at − 20 °C until needed.

##### Quantitative analysis of ADA skin distribution from the prepared gels using UPLC

Ex vivo skin deposition of the prepared ADA-OPTMS gel and ADA-gel was evaluated using the tape stripping method utilizing mice dorsal abdominal skin excised as a diffusion membrane. The skin was defrosted, equilibrated with pH 7.4 phosphate buffer solution, and then fitted to a Franz diffusion cell with a 3.14 cm^2^ diffusional area^31^. To maintain sink condition, 20 ml of a 60:40 v/v mixture of phosphate buffer (pH 7.4) and THF was added to the receptor compartment. This mixture was magnetically agitated, and the temperature of the circulating water bath was maintained at 37 ± 1 °C. The donor compartment was mounted with 0.5 g of ADA-OPTMS gel or ADA-gel. Samples were taken from the receptor compartment after 24 h, and the unpermeated drug was then removed by washing the upper part of the skin five times with distilled water.

Then, the tape stripping technique was performed starting with the stratum corneum that was separated using twenty strippings of a Scotch adhesive tape. Next, the dermis was shed from the epidermis utilizing forceps. Each membrane was chopped and soaked overnight in tetrahydrofuran to completely extract ADA. All samples, including the 24-h sample from the donor compartment, were filtered using a 0.44 µm syringe filter and analyzed. All experiments were conducted in triplicate^30^.

The concentration of Adapalene in each layer was evaluated by ultra-performance liquid chromatography (UPLC) method as stated by Najafi-Taher et al., 2018, with slight modifications. Briefly, the isocratic elution of the UPLC analysis method was performed using a C18 column (100 mm × 4.6 mm, particle size 3 μm) and 100% acetonitrile as a mobile phase with a flow rate adjusted to 0.6 ml/min, together with an injection volume of 10 μl and a temperature of 35 °C. For the detection wavelength, lambda was set at 235 nm^32^. Furthermore, the UPLC method's precision, linearity, accuracy, detection limit, quantification limit, and ruggedness were all validated in accordance with ICH criteria^33^.

##### Qualitative tracing of ADA skin distribution from the prepared gels using confocal laser scanning microscopy

Dil fluorescent dye was selected as the simulated tracer (Zeiss LSM 710, Germany in conjunction with ZEN 2009 software, excitation wavelength: 543 nm, emission wavelength: 633 nm) to visualize the transcutaneous pathway following epicutaneous application of ADA-OPTMS gel and ADA-gel. Using the same preparation technique as previously described, fluorescent dye “Dil” partially replaced the ADA loaded into the microsponges of the ADA-OPTMS gel, and ADA in the reference gel (ADA-gel) to produce the fluorescently tagged formulae. As previously mentioned in the quantitative ex vivo deposition investigation, the labeled formulae were placed on the donor compartment of the Franz diffusion cell for 24 h. After that, the skin pieces were taken out of the diffusion cells, washed, and put away for further manipulations essential for visual inspection^34^.

#### In vivo evaluation of the prepared ADA-OPTMS gel using an acne mice ear model

##### Bacterial culture and animals

Standard *Propionibacterium acnes* strain ATCC 6919 was cultivated anaerobically on RCM broth for 48 h at 37 °C until the OD 600 nm value was equal to one, which translates to 5 × 10^11^ CFU/mL. This bacterial suspension served for the purpose of inoculum preparation^35^.

In the current investigation, 30 male BALB/c mice (immunocompromised strain) weighing 20–30 g (obtained from the animal house of the Faculty of Pharmacy—The British University in Egypt) were employed. Randomly assigned animals were housed at a temperature of 22 °C with a humidity of 5%, 12 h of light (light starts at 8:00 a.m.) and 12 h of darkness, given unlimited access to standard water and food in the lab.

The study was performed after the approval of the research ethics committee for experimental and clinical studies of the Faculty of Pharmacy, Cairo University-Cairo-Egypt (no. PI-2287). Additionally, in accordance with the guidelines of the "Guide for the Care and Use of Laboratory Animals" and the protocol of the Helsinki agreement Additionally, the study is reported in accordance with ARRIVE guidelines and it complied with local institutional legislation for the protection of animals under the direction of qualified examiners, as well as Egyptian laws on animal protection.

##### Induction of inflammatory acne model in mice ear

The cultured *P. acnes* cells were collected, and the concentration was adjusted to 1.0 × 10^7^ CFU per 20 µL in saline for each injection. The right-side ears of Balb/C mice were intradermally injected with the living *P. acnes* suspension to induce inflammatory acne in the animals. After being left for 24 h, the microcomedone^36^ shall become visible and will be validated by an average 0.1 mm rise in ear thickness as measured by a Vernier Calliper^37^.

##### Experimental design

After the induction of the microcomedone, the mice were randomly split into two studies as follows:

*The first study*

Mice were randomly divided into three groups, each consisting of six animals. The groupings were as follows. Group A: The mice infected in the right ear received no treatment and acted as a model group. Group B: The mice infected right ear were rubbed with 0.1 g of marketed Adapalene 0.1% gel twice daily, acting as the market product group. Group C: The mice infected right ear were rubbed with 0.1 g of ADA-OPTMS gel (0.1% ADA) twice daily, acting as the test group.

Five doses were administered over the course of the experiment's three consecutive days. Specifically, on the first day, a single dose was applied upon reaching the targeted size of the infected ear, whereas the subsequent days followed a twice-daily dosing schedule.

Additionally, the difference between the size of the infected ear before injection and 24 h after injection was calculated using a Vernier calliper to determine the size of the formed microcomedone in each group. Additionally, measurements of ear thicknesses 24 h, 48 h, and 72 h after the treatment start were made, and the differences were computed in a similar manner. Each dimension was measured in triplicate. The findings are presented as the mean ± SD for different groups. Additionally, daily digital photos of the condition were taken both before and after therapy to track the development of inflammation^35,38^.

*The second study*

Twelve animals were used for this study to evaluate the timeline effectiveness of the treatment with ADA-OPTMS gel (0.1% ADA). The same methodology mentioned before (in first in vivo study section) was applied for dosing and application of treatment. Six mice were mercifully sacrificed after 1 day of treatment (total one dose applied) and the other six after 2 days of treatment (total three doses applied), solely for histopathological examinations.

##### Histopathological evaluation

At the conclusion of the in vivo studies, neck dislocation method was used to mercifully sacrifice every animal before being dissected. All of the infected groups' autopsy samples, along with the healthy, uninfected ears, were chopped and kept in neutral formalin (10%) for about 72 h. After that, the dissected samples were given an ethanol and xylene treatment before being injected with artificial paraplast tissue embedding media. To display various skin layers, tissue segments from the central zones of several auricular samples were cut using a rotating microtome. After that, tissues were mounted on slides, stained with hematoxylin and eosin, and then inspected with the aid of a microscope by a trained histologist utilizing a Full HD microscopic imaging system (Leica, Germany)^39,40^.

The chronicled lesions were scored by the scoring system adopted by Abdelkader et al., 2021, which was (−) for no lesions present, (+) mild lesions comprising 15% of the examined tissue sections, (++) moderate lesions noted in 16–35% of the tissue sections and (+++) more than 35% lesions found in examined tissue sections indicating high severity^41^.

##### Histochemical analysis

To conduct immunohistochemical analysis, deparaffinized retrieved auricular tissue sections from the first in vivo study were subjected to 0.3% H_2_O_2_ treatment for 20 min. After that, samples were incubated with an anti-p-NFκB p65 antibody (GTX54672, GeneTex Inc.—1:100) overnight at 4 °C. After being washed with phosphate buffered saline pH 7.4, tissue sections were then treated with the secondary antibody from the HRP Envision kit (DAKO) for an additional 20 min, followed by a second wash out and an additional 15 min of incubation with the dye diaminobenzidine (DAB). The slides were then cross stained with hematoxylin, dehydrated, and cleaned in xylene before being covered for microscopic analysis following a final PBS wash. For the purpose of determining the relative area % of immunohistochemistry expression levels of phospho-NF-κB in dermal/epidermal cellular elements, six nonoverlapping fields were arbitrarily nominated and perused from each sample^40,42^.

#### Clinical study of the prepared ADA-OPTMS gel

##### Settings

Under the direction of Minia University's Postgraduate Studies and Research Ethical Committee, the current investigation was conducted in the dermatology outpatient clinic of the hospital. The panel for experimental and clinical studies at Cairo University's faculty of pharmacy accepted the study protocol as well (no. PI-2287), and the research complied with the Helsinki Declaration's ethical principles. Before participating in the trial, patients provided written informed consent.

A total of ten patients (age ≥ 16 years) with mild and moderate acne vulgaris with reference to the Acne Consensus Conference (ACC) participated in the current double-blinded, randomized, split-face, preliminary clinical study. The exclusion criteria were as follows: receiving any acne treatment, whether topical or systemic, in a 3-month period before examination required to be enrolled in the study, pregnant or lactating females, history of allergies or sensitivity to topical retinoids, history of receiving any systemic antibiotics by month prior to the start of study and deep facial cleansing or laser treatment within a 1-month period prior to the trial.

##### Treatment protocol

The prepared ADA-OPTMS gel (0.1% ADA) formula and the market product Adapalene gel (0.1%) were given code names DAP and MAP, respectively. Those codes were unknown for both physicians and patients. Also, each container was labeled either left or right to avoid any confusion by the subjects during the study. Each patient was instructed to softly cleanse their face and apply a thin film of DAP on the right side and MAP on the left side of their face, only on the affected areas with caution to avoid contacting eyes or lips, once at night^43^. Additionally, they were instructed to wash their faces in the morning and to minimize sun exposure, with application of sun block if feasible. The duration of the treatment lasted for a period of 12 weeks^44^. The dermatologists performed biweekly follow-up outpatient visits to monitor the treatment and for any reported adverse events.

##### Primary clinical assessment

Patients were mainly photographed and assessed at baseline (start of treatment) and at the end of the treatment period (after 12 weeks). The evaluation of treatments was determined via reckoning the number of lesions of various types present on each side of the face, which was conducted blindly by proficient dermatologists. The lesions were either inflammatory (papules, pustules or nodules) or noninflammatory (open and closed comedones). The posttreatment percent reduction in the number of noninflammatory, inflammatory, and total lesions was calculated at the final assessment, where the number of lesions at baseline was assumed to be 100% and the reduction percent was calculated accordingly^45^.

##### Tolerability assessment

The irritation potential of DAP and MAP was evaluated during the biweekly follow-up visits. The evaluation was conducted through interviewing the patients about any treatment-associated side effects, including erythema, pruritus, scaling, and burning sensation. A 4-point scale was used for the assessment as follows: 0, no seeming side effects; 1, mild; 2, moderate; or 3, severe side effect(s)^46^. Additionally, any adverse events were spontaneously reported for safety measures^47^.

##### Secondary clinical assessment

The secondary efficacy measures were the physician’s global assessment (PGA) and patient’s global assessment (PaGA). PGA was conducted at the start and end of the treatment for each face side for each patient. The PGA comprised of a 5-point scale, in which 0 denotes clear or minor residual hyperpigmentation or erythema; 1 denotes nearly clear skin or a low number of dispersed comedones and few papules; 2 denotes the presence of a mild degree of acne; 3 refers to the presence of lesions with a moderate degree or many comedones, papules, and pustules; and 4 reflects the presence of a severe degree of acne, with a large number of comedones, papules, and pustules (also a few nodules and cysts may be present)^48^. Concurrently, PaGA was performed for the satisfaction of the patient on the overall treatment on each side of the face. The physician questioned the patients regarding the spreadability, absorbability, how it felt on the skin, efficacy, dryness and irritation, and overall satisfaction. The survey was divided into four categories: dissatisfied, slightly satisfied, moderately satisfied and fully satisfied^49,50^.

### Statistical analysis

GraphPad Prism 8 software (San Diego, CA, USA) was utilized for the statistical analysis of the ex vivo and in vivo studies^51^. The results of the quantitative skin deposition were analyzed using two-way ANOVA. The variation in the recorded pixels using CLSM was also analyzed via two-way ANOVA, followed by Sidak's multiple comparisons post hoc test. Regarding the in vivo investigation, two-way ANOVA was used to analyze variations in the mean values of the infected ear before and after therapy for each group followed by Tukey's and Dunnett's post hoc tests. Accompanied by the analysis of NF-κB levels using one-way ANOVA followed by Tukey's multiple comparison test.

For the clinical study, the statistical analyses were executed using SPSS package software for Windows, version 24.0 (SPSS Inc., USA). The before and after treatment data were analyzed using the Wilcoxon signed rank test for nonparametric quantitative data. The analysis of the difference between treatments on both sides was carried out by the Mann‒Whitney test for nonparametric quantitative data. The qualitative data between the two sides were evaluated using Fisher exact test. In all the performed statistical analyses, *p* values ≤ 0.05 were considered significant.

### Ethics approval

This study was performed in line with the principles of the Declaration of Helsinki. Also, the study is reported in accordance with ARRIVE guidelines. Approval was granted by the Ethics Committee of experimental and clinical studies of the Faculty of Pharmacy, Cairo University-Cairo-Egypt (no. PI-2287).

### Consent to participate

Informed consent was obtained from all individual participants included in the study.

### Consent to publish

The authors affirm that human research participants provided informed consent for publication of the images in Fig. 8.

## Results and discussion

### Design analysis

To obtain the finest MS system, having the highest P.Y.%, E.E.% and optimum particle size with maximum cumulative drug release, BBD was applied. As shown in Table 2, fifteen formulae were successfully prepared using the quasi-emulsion diffusion method with variations in Eudragit RS100 percent in polymer mixture (EUD%) (A), the volume of organic phase (B) and the drug to polymer mixture percent (drug%) (C). Meanwhile, the following parameters were fixed according to previous screening results^17^: the amount of emulsifying agent, sonication time, method of organic phase addition and stirring speed. The efficient preparation of porous MS and competent drug entrapping was also confirmed by SEM imaging, as shown in Figure S1.

**Table 2:** Composition of the suggested ADA-MS formulae with the experiential values of dependent responses.

Formulae	Eudragit RS100 percent in Polymer mixture (%)	Volume of organic phase (ml)	Drug to Polymer mixture percent %	Y1 (%)	Y2 (%)	Y3 (µm)	Y4 (%)
F1	100	25	75:25	85.63 ± 2.6	84.6 ± 2.4	14 ± 2.11	42.19 ± 4.2
F2	50	20	75:25	92.1 ± 2.54	82.7 ± 0.34	21.2 ± 3.89	65.41 ± 3.5
F3	0	15	75:25	93.5 ± 3.32	88.55 ± 0.8	41.3 ± 4.71	56.9 ± 3.72
F4	100	15	75:25	73 ± 2.7	82.7 ± 3.6	28.8 ± 1.27	50.76 ± 3.89
F5	100	20	80:20	78.3 ± 1.78	78.18 ± 1.8	22.8 ± 0.40	53.68 ± 3.63
F6	0	25	75:25	90.3 ± 2.1	81.15 ± 0.54	35.5 ± 4.46	27.1 ± 4.45
F7	50	20	75:25	89.9 ± 1.89	73.6 ± 0.63	25.6 ± 1.47	61.1 ± 2.6
F8	50	25	80:20	97 ± 1.23	82.68 ± 2.2	19.5 ± 1.53	63.5 ± 2.33
F9	0	20	80:20	98.9 ± 1.45	95.63 ± 1.1	39.5 ± 3.04	39 ± 2.17
F10	50	15	70:30	99.4 ± 0.8	99.9 ± 0.4	31.3 ± 2.21	76.9 ± 1.76
F11	50	15	80:20	93.63 ± 1.02	94.5 ± 1.5	26.9 ± 2.47	42.3 ± 1.5
F12	0	20	70:30	90.5 ± 1.56	84.17 ± 3.2	33.9 ± 3.06	33.5 ± 3.42
F13	50	20	75:25	91.25 ± 1.1	75.1 ± 3.1	17.2 ± 1.54	61.8 ± 1.43
F14	100	20	70:30	89.68 ± 2.34	99.9 ± 0.55	28.9 ± 1	24.9 ± 3.3
F15	50	25	70:30	93 ± 0.7	97.67 ± 0.87	18.6 ± 2.52	25.8 ± 4.1

*Y1* production yield %, *Y2* entrapment efficiency %, *Y3* particle size, *Y4* cumulative drug release after 24 h %

According to Table 3, the suggested models for all responses P.Y. %, E.E.%, P.S. and Q24h were significant and were found to be quadratic. Table S1 shows the models values of lack of fit, correlation coefficients, R^2^ and adequate precision which emphasize the reliability of the model to navigate the design space, together with BBD generated polynomial equations for each variable.

**Table 3:** ANOVA of the dependent parameters (Y1–Y4).

Independent variable	Y1 (%)		Y2 (%)		Y3 (µm)		Y4 (%)	
	C.E	*p* value	C.E	*p* value	C.E	*p* value	C.E	*p* value
Model		0.00363*		0.0166*		0.0131*		0.005*
A	− 5.83	0.001*	− 0.50	0.720	− 6.96	0.002*	1.88	0.365
B	0.80	0.347	− 2.46	0.122	− 5.09	0.008*	− 8.53	0.006*
C	− 0.59	0.476	− 3.86	0.033*	− 0.50	0.689	4.67	0.056
AB	3.96	0.015*	2.33	0.269	− 2.25	0.234	5.31	0.103
AC	− 4.94	0.006*	− 8.32	0.007*	− 2.92	0.139	5.82	0.081
BC	2.44	0.075	− 2.37	0.260	1.33	0.462	18.08	0.001*
A^2^	− 5.94	0.003*	1.45	0.490	7.88	0.006*	− 16.44	0.002*
B^2^	0.47	0.697	5.67	0.033*	0.68	0.709	− 2.09	0.486
C^2^	4.21	0.014*	10.91	0.003*	2.06	0.288	− 8.56	0.027*

C.E.: Coefficient estimate, A: Eudragit RS100 percent in polymer mixture, B: volume of organic phase, and C: Drug to polymer percent. Y1: Production yield %, Y2: Entrapment efficiency % Y3: Particle size and Y4: Cumulative drug release after 24 h% * Significant *p* value < 0.05.

**Figure 1:**
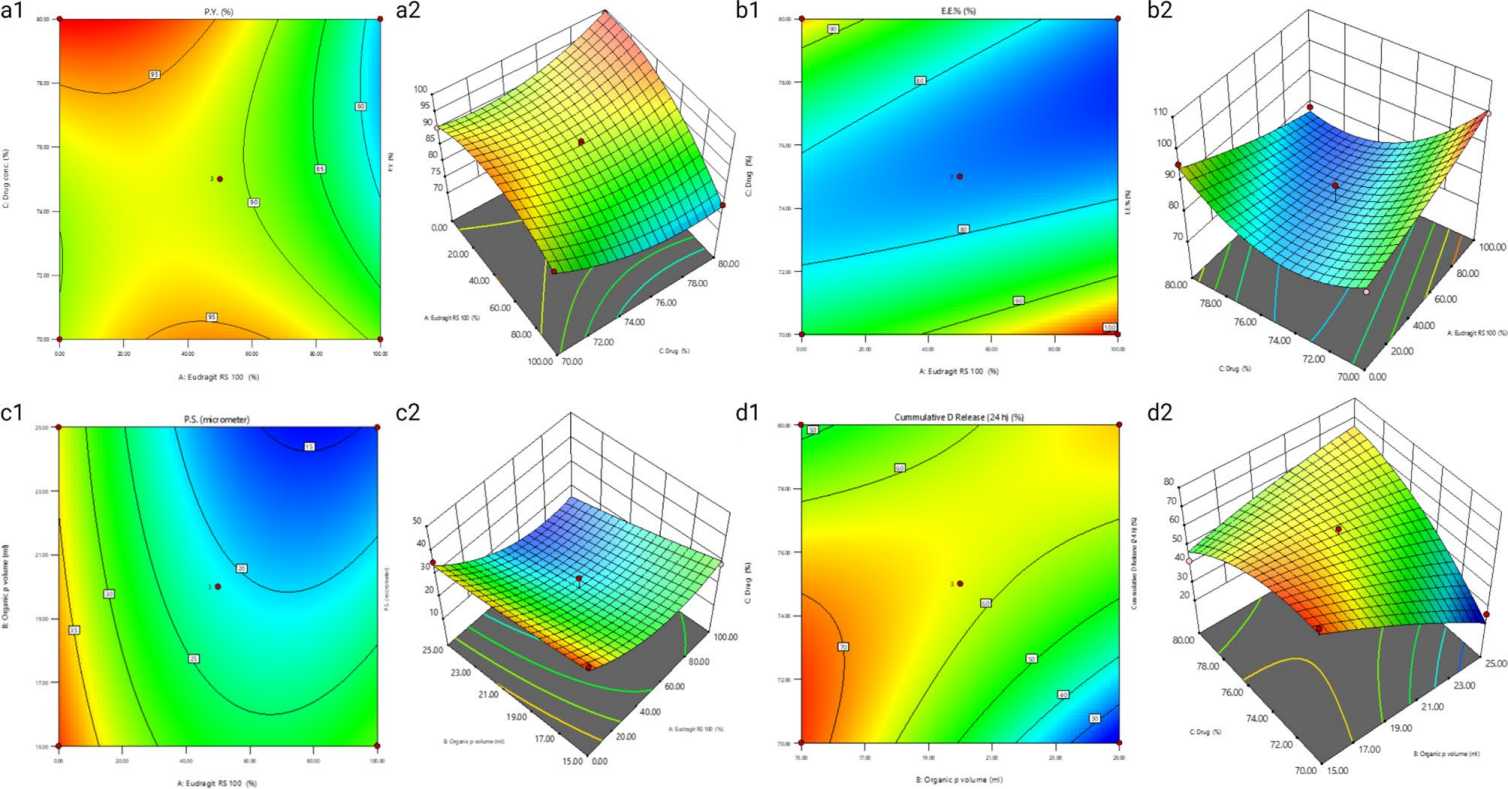
The Box–Behnken design response surface plots 1: contour plots and 2: 3D plots of (**a**) production yield percentage, (**b**) entrapment efficiency percentage, (**c**) particle size and (**d**) cumulative percent release after 24 h.

Table 3 and Fig. 1 show the results of the statistical analysis, contour plots and three-dimensional response surface plots of the effect of independent variables on the dependent ones for the prepared ADA-MS formulations.

#### Analysis of the influence of independent variables on the production yield (Y1)

Achieving the upmost production yield is essential in the pharmaceutical industry to ensure maximum return. The results clearly depict that the Eudragit RS100 percent in polymer mixture (EUD %) (A) had a significant antagonistic effect on the P.Y.% of the prepared ADA-MS. This could be justified by the effect of polymers on the microenvironment viscosity, where an adequate viscosity is required to retard the diffusion of the organic phase into the aqueous phase. If the diffusion process occurs rapidly, the drug and polymers precipitate prior to globule formation, and thus, MS are not formed^52^. Additionally, it has been previously reported that both EUD and EC are film formers and are capable of increasing the viscosity of organic media; however, it was found that at the same polymer concentration, EC increased the viscosity of the media 3.5-fold more than EUD^53^. In our study, an increase in EUD% coincided with a decrease in EC% within the polymer mixture, primarily due to its influence on viscosity and droplet formation. Consequently, the rise in EUD%, in relation to EC%, resulted in a decreased overall P.Y.%.

Alongside, the interaction between the Eudragit RS100 percent in polymer mixture (EUD%) and drug to polymer percent (drug%) (AC) had an antagonistic significant effect on P.Y.%. This could pertain to the increase in the drug ratio synchronically decreasing the polymer ratio, and thus, as explained before, the globule stability was compromised due to the presence of a low polymer percentage, especially EC%, leading to lowering of the P.Y.%^52^.

On the other hand, the interaction between EUD% and the organic phase volume (AB) had a significant agonist action on P.Y.%. However, the low EUD % with a low volume of organic phase (DCM) had a high P.Y.% due to the formerly mentioned effect on viscosity.

Yet, increasing the EUD% with increasing DCM had a cooperative positive effect, in which the aforementioned inharmoniousness between the EUD% and droplet formation could have been resolved by increasing the solvent quantity, and thus, the time required for evaporation increases, allowing enough time for MS formation and increased P.Y.%^52,54^.

#### Analysis of the influence of independent variables on the entrapment efficiency (Y2)

Entrapment efficiency % is one of the key characteristics that affects formulation selection, and obviously upmost drug loading is required, resulting in piercing investigations on the factors affecting such property. According to Table 3, the drug to polymer mixture percent (drug%) (C) showed a significant inverse effect on the E.E.% of the prepared ADA-MS. This could be interpreted by increasing the drug ratio relatively decreases the polymer ratio. This leads to a reduction in the number of pores and the availability of space to accommodate the drug moiety; consequently, poor encapsulation occurs^21,25,55^. Also, the interaction of EUD% with drug% (AC) had a highly antagonistic significant effect on E.E.%. This could be elucidated according to the previously clarified effect of polymer-induced viscosity on the droplet formation process.

#### Analysis of the influence of independent variables on the particle size (Y3)

Particle size is one of the aspects that impacts the topical delivery of the active moiety and its entrapment within the skin layers. It was observed that the Eudragit RS100 percent in polymer mixture (A) had a significant antagonistic effect on particle size. EUD has been previously reported to form a more flexible polymer coat than EC^53^, consequently increasing EUD%, leading to the formation of ADA-MS particles of smaller particle size. Another explanation is that the EUD, as previously mentioned, has a lesser effect on the viscosity of the organic phase, and as a result, a less viscous medium is formed, which requires low energy to divide into globules with a small diameter, resulting in the formation of ADA-MS with a small particle size^18,56^.

Likewise, organic solvent volume (B) had a significant antagonistic effect on P.S., which was consistent with the previous findings relating the effect of the factor on the microenvironment viscosity. The decrease in the DCM volume origins a highly viscous microenvironment, which requires high energy to divide and eventually forms bulky globules and yields MS of large size^20,57,58^.

#### Analysis of the influence of independent variables on the cumulative drug release after 24 h % (Y4)

*The* in vitro release profile of the drug from all fifteen formulae was performed and plotted in Figure S2. The cumulative drug release after 24 h was one of the vital factors desired to be monitored and employed by the BBD to elucidate the optimum formula. It was demonstrated that the organic phase volume (B) had an inverse significant effect on Q24h. This may be attributed to the abundance of a large quantity of DCM, which contributed to the formation of MS with a highly porous and spongier structure^59^. Additionally, it is worth mentioning that voids present in microsponges have been reported to act as reservoirs for drugs and are accountable for delaying the drug release process^55^.

The interaction between the organic phase volume and drug-to-polymer mixture percent (BC) had a significant positive effect on Q24h. Whereas, increasing the DCM volume with increasing drug% led to an increase in Q24h, and this feature could be explicated by the presence of a large volume of DCM, with a large quantity of the drug, depositing the drug moiety at the peripheral layers of the microsponges^60^. Then, again, increasing the volume of the organic phase with a decrease in drug% led to a decrease in Q24h, which is logical due to the presence of a lower drug amount in conjunction with a higher amount of polymer that forms a thick polymer wall of MS, which lengthens the diffusion path, making it difficult for the drug dissolution process and thereby resulting in a slower release rate^20,61,62^.

### Optimum formula selection and evaluation

To optimize the ADA-MS formula, constraints were applied to maximize P.Y.%, E.E.% and Q24h while maintaining the particle size in range. The desirability of the optimized formula was 0.938 (nearest to 1). The optimized formula, entailed 43.1% Eudragit RS100, 15 ml DCM and 70% drug, was prepared, and the responses were carried out in order to confirm the validity of the calculated optimal parameters and the predicted responses.

The adapalene-loaded microsponges optimized formula (ADA-OPTMS) achieved 98.3% ± 1.66% P.Y.%, 97.3% ± 1.64% E. E% and a particle size of 31.8 ± 1.1. As for the in vitro release profile shown in Figure S3 revealed that ADA was released in a biphasic pattern with an initial burst of 19.9% ± 1.47 release within the first hour, followed by sustained release for 24 h, reaching 75.1% ± 1.4 cumulative release. However, the free drug exhibited a higher and faster release than from the prepared ADA-OPTMS formulation. The free ADA exhibited about 35% ± 2.33% cumulative release after 2 h. After 6 h, the total ADA amount was mostly released (approximately 97.3% ± 2.56) from the membrane. This noteworthy difference in ADA release behavior in both cases might be explained by the fact that the release of ADA from the microsponges occurred in a more controlled manner. This could be owing to the formation of effectual MS with adequate pore size and ADA being homogenously distributed between the matrix layers. The drug adsorbed to the surface of MS was released within the first hour, which ensured the onset of action, followed by matrix release that ensured sustainability^63^.

The predicted values of the dependent parameters were assessed in relation to the observed ones and the percent bias was estimated as shown in Table S2. It was eminent that the observed values were in agreement with the predicted values, and the bias % values were found to be within 5%, indicating the efficiency and validity of the BBD for the optimization process^64^.

**Figure 2:**
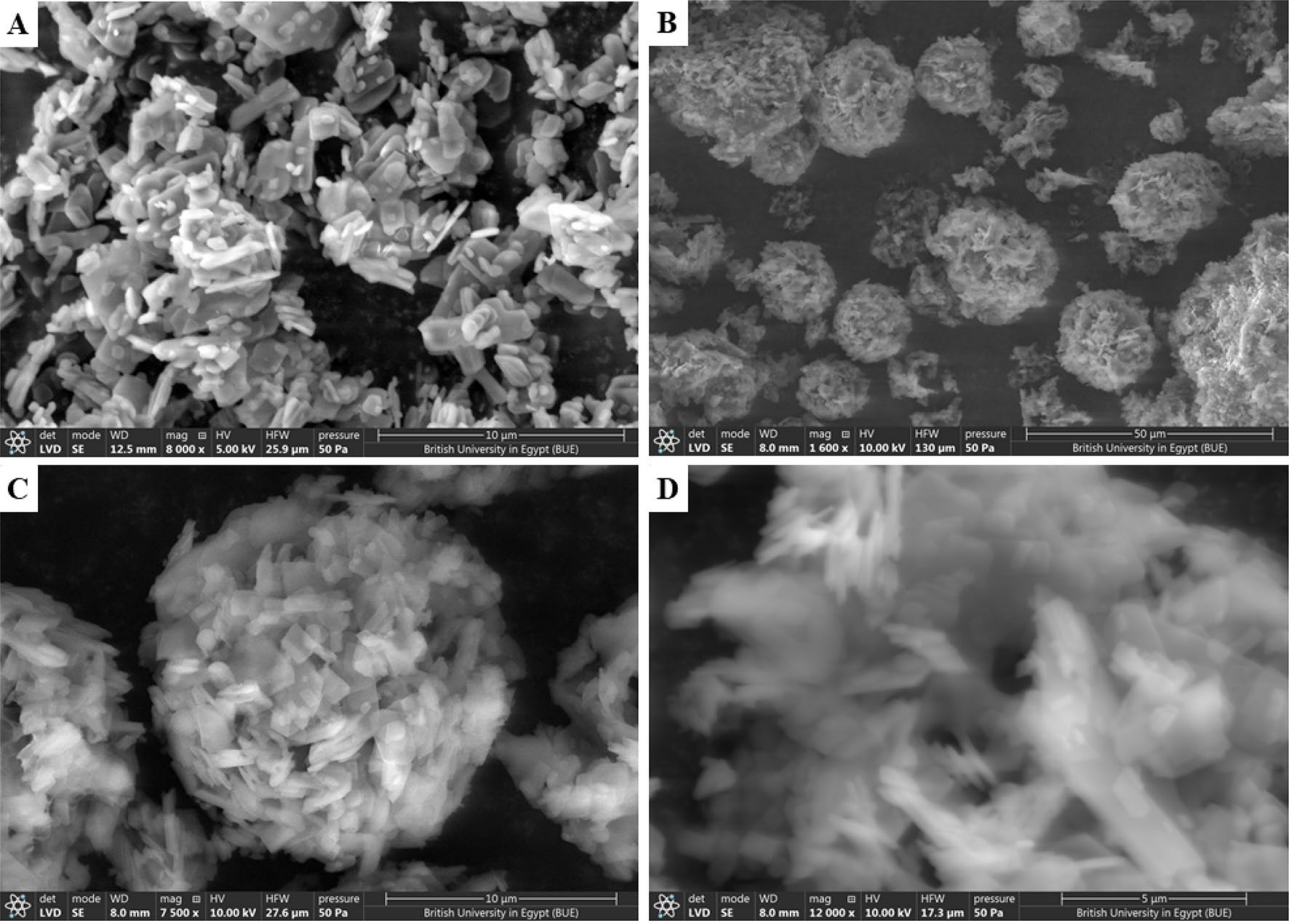
Scanning Electron Microscope images of (**A**) Adapalene pure, (**B**–**D**) optimized adapalene-loaded microsponges formula (scale 50 µm, 10 µm and 5 µm, respectively).

Visualization by SEM imaging was achieved (Fig. 2), primarily microphotographs for the pure adapalene crystals were captured, as well as the optimum formula at different magnifications. To display the distribution of MS, ADA loading, pores and overall MS shape. The images reflected the presence of numerous homogenous porous spherical microsponges particles with ADA crystals located both on the surface and the core of the MS particles. This indicated the successfulness of preparation of porous MS via the evaporation of DCM and competent drug entrapment^65,66^.

### Preparation of gel containing ADA-OPTMS

With the intention of topically applying ADA-OPTMS, it was incorporated into a gel base. Gels as a topical vehicle have been reported to provide a cosmetically elegant dosage form that forms a greaseless film with ease of application and removal. It was especially recommended for oily and hair-bearing areas, which is convenient for acne treatment^67^. The ADA-OPTMS gel was successfully prepared. The selected gelling agent was Carbopol 934 polymer, which is widely used in topical formulations and has excellent stability. Additionally, the penetration enhancers propylene glycol and ethanol were added with the aim of augmenting the percutaneous penetration of ADA^68^. Furthermore, the application of gels involves rubbing, which causes pressure that triggers the initiation of drug release from the microsponges^15,69,70^.

### Characterization of the prepared ADA-OPTMS gel

#### Physical examination

It is clearly evident that the prepared ADA-OPTMS gel was odorless and had acceptable presentation with a whitish, smooth and homogenous appearance. These findings warrant patient compliance upon usage of the prepared gel^71^. These results were found to be consistent with the findings of another study conducted by Nagula et al., 2020, where microsponges-based gels of naringenin were prepared^56^.

#### The pH

The pH value of the ADA-OPTMS gel formula was found to be between 6.9 ± 0.09, which was within the acceptable range for topical application to avoid dermal irritation^32^. Thus, the prepared gel was considered suitable for dermal application.

#### Spreadability

The spreadability was considered a vital property for gel formulations to ensure the ease of application with an adequate surface area for drug permeation. The spreadability of the ADA-OPTMS gel was found to be 4.6 ± 0.3 cm, which is within a suitable range for reasonable spreadability^26^.

#### Rheological study

The rheological properties of the ADA-OPTMS gel, as shown in Figure S4, demonstrated an indirect correlation between shear rate and viscosity in which an increase in the first led to a decrease in the latter with an elevation in shear stress. As the shear stress increases, the molecules of the gelling agent align their long axes in the flow way, and such alignment minimizes the internal resistance of the gelling agent, which subsequently causes a reduction in viscosity. These findings line up with the non-Newtonian pseudoplastic flow with shear thinning behavior^26,30^. The shear thinning behavior was presumed to be optimum for topical application, as the rubbing resembles the shear force, and thus, an enhanced spreading of the formula takes place^37^.

#### Drug content

The percent of ADA recovered from the ADA-OPTMS gel was 97.6% ± 0.4. The drug content of ADA-loaded MS in the gel base was lucrative, as the value did not stray from 95%^29^. Furthermore, the low value of S.D. (coefficient of variance % = 0.0041) ensured the consistent dispersion of ADA within the gel formula^26^.

### Ex vivo skin deposition study for the prepared gels

#### Quantitative analysis of ADA skin distribution from the prepared gels using UPLC

Mouse skin was selected for the deposition study, as it is archetypal to human skin, to assess the ADA skin distribution from the prepared ADA-OPTMS gel and ADA-gel. In the current study, the deposited and permeated amounts of ADA from the prepared ADA-OPTMS gel in comparison with the reference gel (ADA-gel) were calculated using the UPLC analysis method. The UPLC assay of ADA in phosphate buffer pH 7.4–THF (60: 40 v/v) using the isocratic method had a good retention time of 4 ± 0.5 min. The method was considered valid, as the calculated parameters did not stray from the acceptable ranges reported in the guidelines^33^, as shown in Table S3.

**Figure 3:**
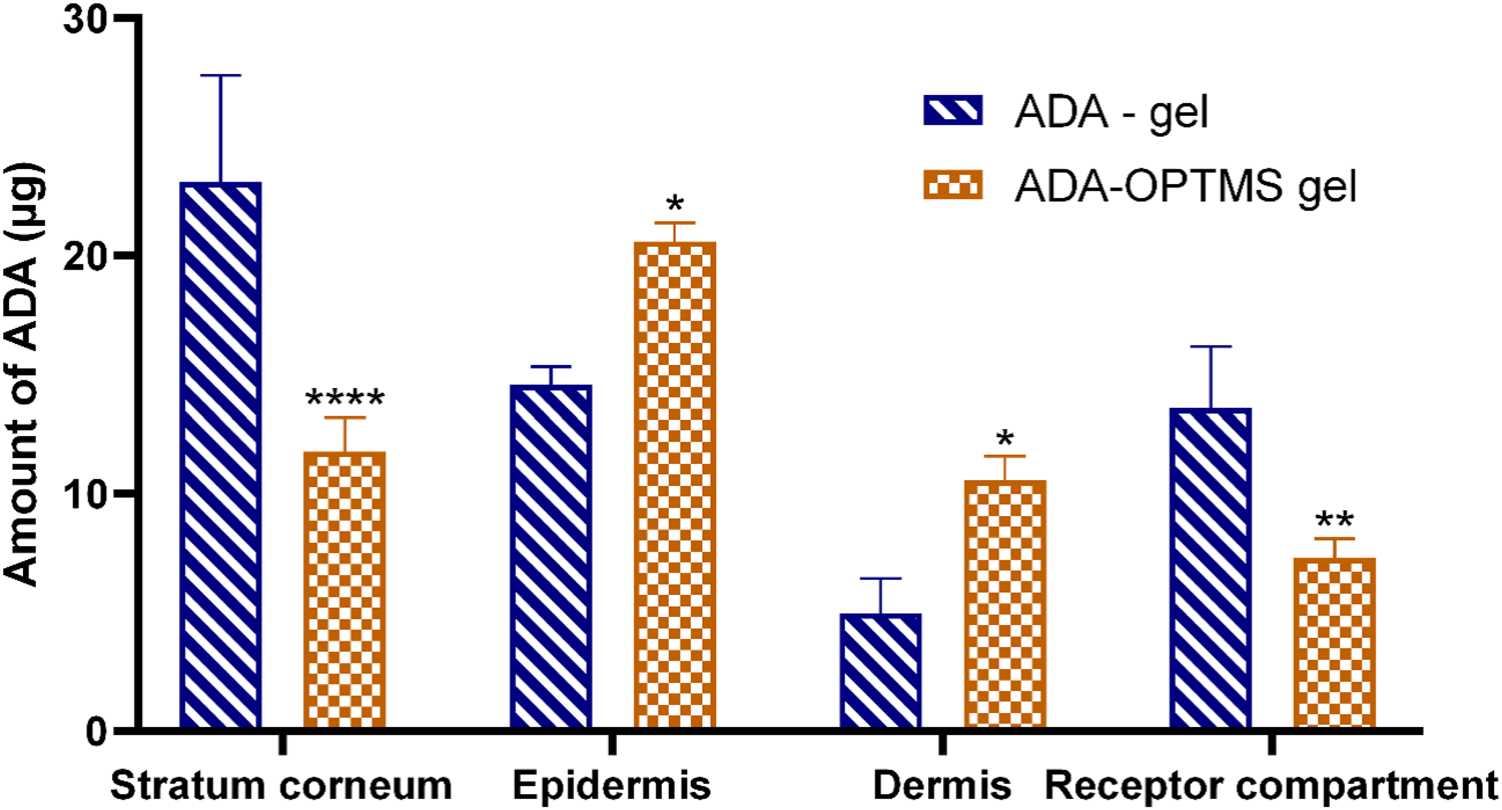
Deposited amount of adapalene from the ADA-gel and ADA-OPTMS gel in each skin layer and in the receptor compartment after 24 h. The results were compared using two-way ANOVA followed by Sidak's post hoc test. *p < 0.05; **p < 0.01; ****p < 0.0001 compared with the respective amount of ADA deposited from the ADA gel.

Figure 3 shows the deposited and permeated amounts of adapalene from the prepared ADA-OPTMS gel and ADA-gel. The results revealed that the amounts of ADA accumulated in both the epidermal and dermal layers by the ADA-OPTMS gel were significantly superior to those deposited by the ADA-gel. It was also observed that the maximum deposited amount of ADA by the ADA-OPTMS gel was in the epidermal layer. Furthermore, the amount permeated after 24 h from the ADA-OPTMS-gel was significantly lower than that from the ADA-gel. For the skin layers, it was clear that the amount retained in the stratum corneum (SC) by the ADA-OPTMS gel was significantly lower than that by ADA-gel.

These results shed light on the importance of the carrier system in targeting the site of action, the pilosebaceous unit found in the epidermis and dermis. For instance, in our study, although the total amount permeated and deposited in all skin layers from both the ADA-OPTMS gel and the reference gel was more or less the same, the pattern of permeation varied greatly with the ADA-OPTMS gel having virtuous targeting capability by accumulating the ADA within the epidermal and dermal layers. Together with low retention in the stratum corneum and minimal systemic access. This may be attributable to the inability of microsponges to pass the skin barrier due to their size; thus, MS persist on the skin exterior with ADA being controllably released over time^14^. The slow release from the MS minimizes the concentration of ADA within the SC, and thus, the diffusion rate into systemic circulation is minimized^13^. Moreover, the high lipophilicity of the MS polymers barred drug diffusion from the skin into the receiver fluid, preserving effective local drug concentration for a prolonged period of time^72^. This also leads to better tolerability of the treatment, as the skin surface is not exposed to the total dose of ADA^73^; henceforth, better patient compliance could be achieved.

Unlike ADA-gel, the presence of large quantities of free ADA in the gel vehicle allowed higher release and partitioning within the SC, increasing the flow toward systemic absorption, with decreased retention in dermal layers^74,75^. Accordingly, lower delivery to the targeted site and increased potential for systemic side effects occur. Moreover, due to the circumstance of the need for multiple applications of the treatment for prolonged periods of time in the management of acne disease^76^, this subjects the skin to increased irritation risk by the effect of high exposure to large quantities of ADA in each application. Along with increased liability to increased systemic absorption with multiple dosing. Accordingly, patient compliance might be jeopardized. Furthermore, recent studies have highlighted the potential benefits of using a less irritating vehicle for topical retinoids, particularly in patients with skin prone to post-inflammatory hyperpigmentation (PIH), as it may contribute to a reduction in PIH^77^.

Thus, the ADA-OPTMS gel was triumphant in predominantly delivering ADA to the site of action, with minimal susceptibility to either systemic side effects or topical irritations. Consequently, patient compliance is more likely to be guaranteed. These results were in agreement with previous studies on ADA particulate drug delivery systems^32,78^.

#### Qualitative tracing of ADA

**Figure 4:**
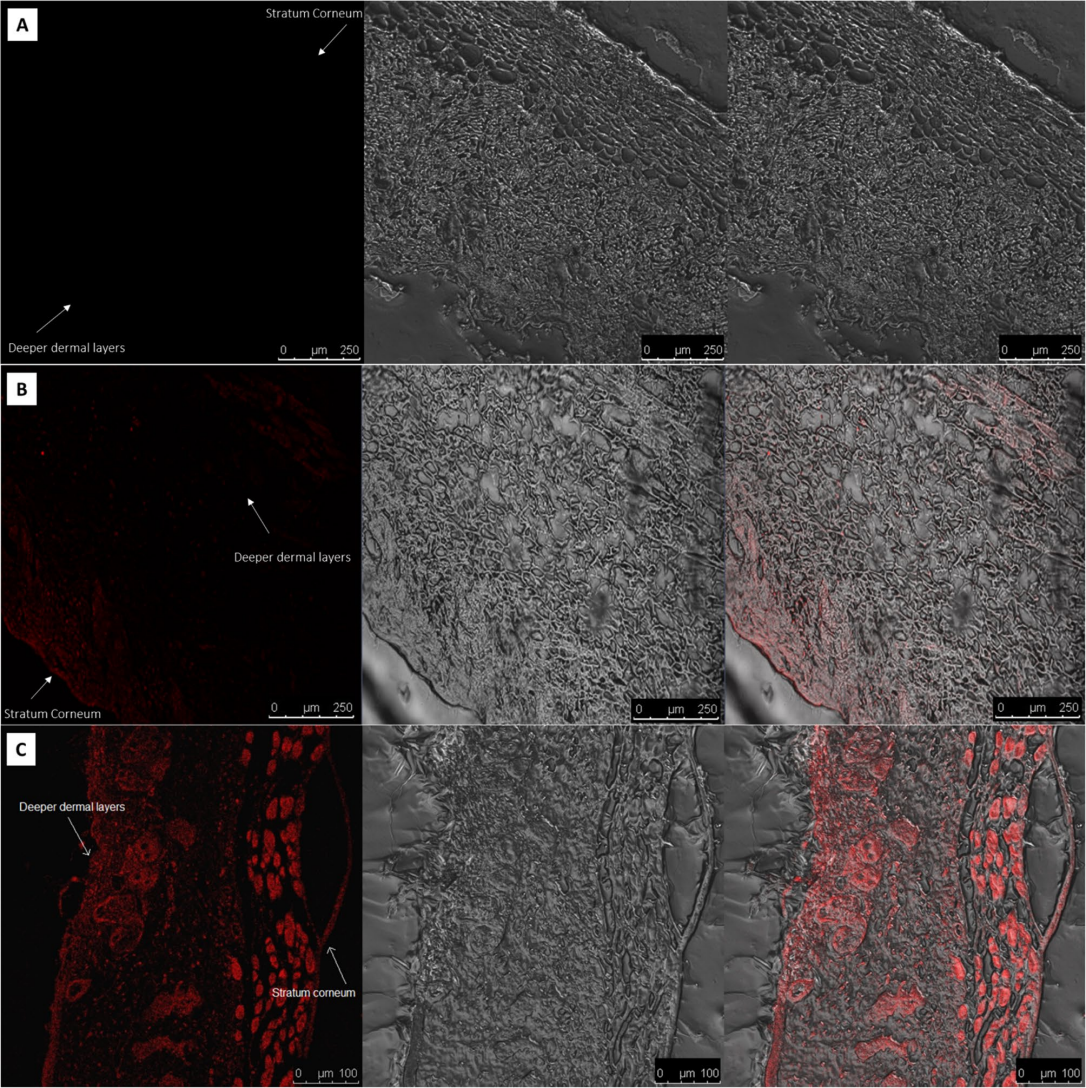
CLSM images showing cross-sectional views of mice skin treated with (**A**) untreated skin, (**B**) Dil fluorescent marker in reference gel, (**C**) Dil fluorescent marker—OPTMS gel, after 24-h application.

As an approach to envisage the delivery pathway and the extent of penetration and accumulation in the skin strata, fluorescently labeled ADA-OPTMS gel and ADA-gel were analyzed using confocal laser scanning microscopy (CLSM). The high lipophilicity of the Dil fluorescent marker (Dil-F) made it an appropriate tracer to imitate the ADA entrapping and delivery behaviors through mouse skin layers. The relative fluorescence intensity of Dil-F within the layers of the skin following the application of fluorescently labeled OPTMS gel and Dil-F loaded into plain gel for 24 h are illustrated in Fig. 4B, C, whereas Fig. 4A represents normal skin to guarantee that there was no autofluorescence. Additionally, Table S4 disclosed the calculated intensities in each skin layer.

It was obvious that the Dil-F-loaded OPTMS gel primarily accumulated in the deeper skin layers: the epidermis and dermis. Additionally, the fluorescence was intensified with high significance (*p* value < 0.0001 and *p* value < 0.005) in the epidermal and dermal layers, respectively, by the application of Dil-F-loaded OPTMS gel over Dil-F-loaded plain gel. Besides, astoundingly, it was vastly distinct that Dil-F was retained in hair follicles. Furthermore, the computed intensities in the stratum corneum from Dil-F-loaded OPTMS gel were significantly lower than those from Dil-F-loaded plain gel (*p* value < 0.05). These results verified the targeting capabilities of the ADA-OPTMS gel to the site of development of acne disease, the pilosebaceous units found in the deeper dermal layers^79^. These findings aligned with the discoveries of quantitative skin deposition testing and reflect the capability of the microspongeal gel to profusely accumulate the drug moiety within the epidermal and dermal layers, specifically the pilosebaceous unit, demonstrating a targeted pattern of delivery. This maybe consequent to the aforesaid hydrophobic nature of ADA and the polymers founding the MS, together with the occlusion effect of the MS, which causes enhanced transcellular penetration, in conjunction with ameliorated trans-appendageal delivery. Furthermore, MS have been reported to be favorably retained at the hair follicle orifice, with their merit in controlled delivery, eventually leading to augmented topical and dermal availability of ADA^29,80^. This study also agrees with the findings of Tolentino et al.^81^, where using advanced carrier systems aided in the targeted delivery of an anti-acne drug into the pilosebaceous units.

### In vivo studies

#### Ear thickness evaluation

*P. acnes* has been acknowledged as the chief bacteria accompanying acne vulgaris pathogenesis in humans. Thus, *P. acnes* has been used in the induction of inflammatory acne vulgaris in the auricles of various mice variant especially Balb/C mice. *P. acnes* elicits inflammatory immune responses when injected into mice ears, causing the development of microcomedones^35^.

**Figure 5:**
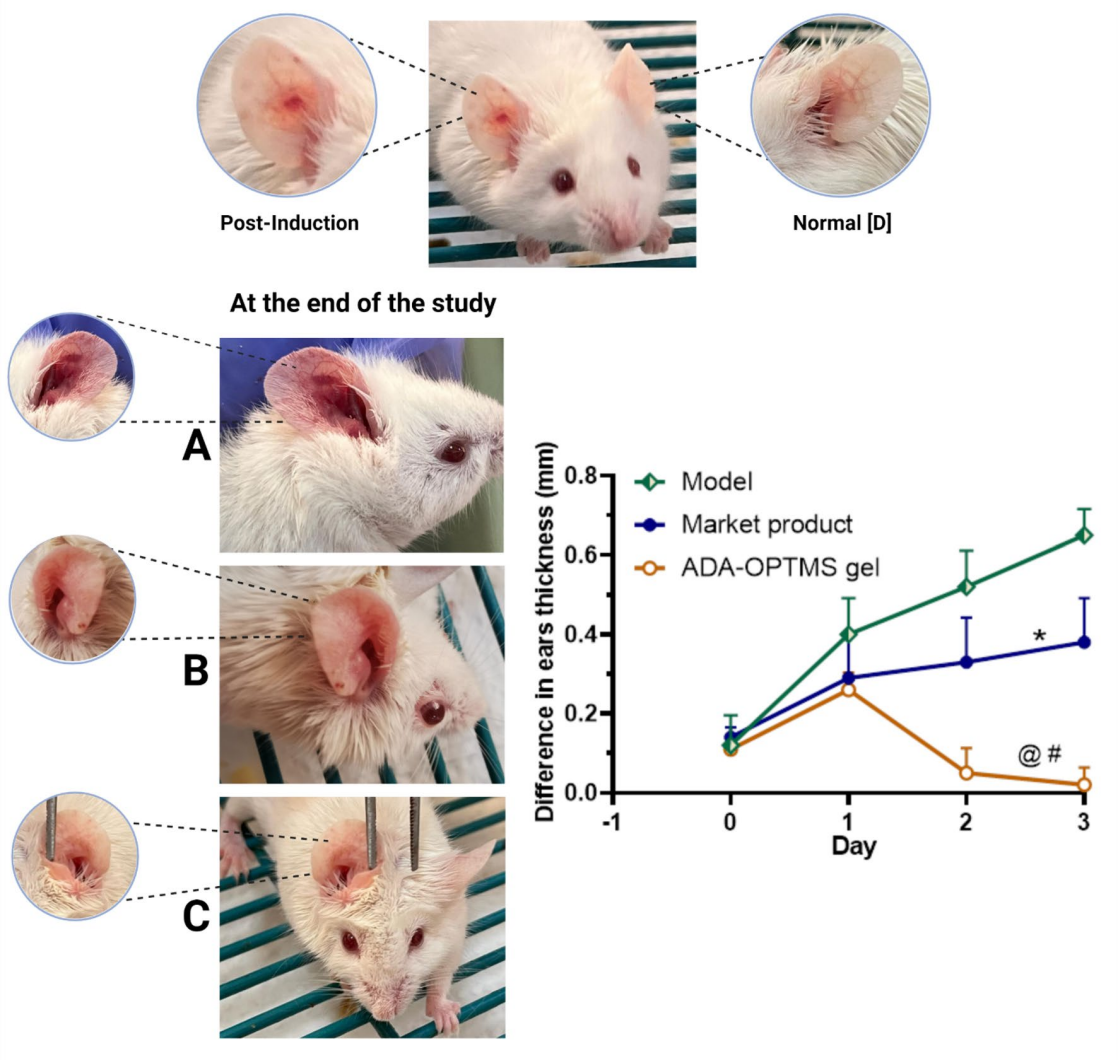
Representative macrophotographs of the mice ear post induction and at the end of the in vivo study and the calculated daily difference in ear thicknesses between the infected ear and the uninfected ear. (**A**) Model ear, (**B**) Market gel Adapalene and (**C**) ADA-OPTMS gel groups (created by Biorender). The results were compared using two-way ANOVA followed by Tukey’s post hoc test (Mean ± SD, n = 6). *p < 0.05 compared with respective model ear thicknesses, ^#^*p* < 0.0001 compared with respective model ear thicknesses, ^@^*p* < 0.01 compared with respective market product Adapalene ear thicknesses.

Visually, microcomedone cysts were assessed 24 h following intradermal *P. acnes* injection in the current investigation. Additionally, all mice had an average 0.1 mm increase in right ear thickness, as shown in Fig. 5. Next, the mice were split in a random manner into groups A, B, and C. Groups B and C were given the therapies that had been nominated, while Group A remained untreated (the model group). The experiment involved applying either Adapalene gel (0.1%) or ADA-OPTMS-gel (0.1% ADA) five times epicutaneously over the course of 3 days.

The ear thicknesses were recorded and photographed daily. The difference prior and post treatments were calculated as shown in Fig. 5. It was palpable that the untreated group A acne progressively developed an inflammatory response during the duration of treatment compared with the normal uninfected ears, recording the highest upsurge in the average difference in the ear thicknesses. Also, it was demonstrated through the variation presented in the macrophotographs compared with the normal ear (Fig. 5D), where the model ear showed distinct cysts formed with swelling, redness and erythema (Fig. 5A), which confirms the success of acne model development.

Moving to the treatment groups, group B received the market product Adapalene gel (0.1%), in spite of the presence of a visible microcomedone (Fig. 5B) by the end of therapy, yet a noteworthy drop in the average difference in ear thicknesses compared with the untreated group A was conveyed, approximately 40% reduction. In consort, group C receiving the ADA-OPTMS gel (0.1%), as represented in Fig. 5C, a marked disappearance of the microcomedone was tangible. Surprisingly, a reduction in the size of the infected ear was detected from the second day of treatment (3 doses applied) with a significant reduction in ear thicknesses (*p* value = 0.002). Furthermore, the ear thicknesses almost matched the normal one by the end of the therapy, with a vibrant significant variance in average ear thickness differences between them and the model group with a *p* value less than 0.0001. Accomplishing practically 97% reduction in ear thicknesses difference.

The average difference in thicknesses between the two treatment groups (B & C) was also found to be of high significance, indicating the superiority of ADA-OPTMS gel in the treatment of acne. Although both treatment groups showed significant differences from the model group, the magnitude of significance of group C was much more dominant than that of group B. This could be explained by the well-known capability of ADA to treat acne disease. In conjunction with the effect of encapsulation into MS, the ADA-OPTMS gel mainly preserved the MS moiety on the skin surface, which had a targeted, occlusive effect with controlled release behavior for ADA^14^.

Additionally, as established in the skin deposition of the present study, the improved ADA penetration and incarceration within the epidermal and dermal layers, aligning with enhanced transependageal delivery^12,13,15,72^, directed the pattern of distribution primarily to the origin of acne disease in the pilosebaceous unit. Likewise, the controlled release manner of the MS well preserved the skin from the irritation side effects of ADA, permitting healing action without aggravation of the inflammatory condition. Furthermore, ADA has been reported to attain an in vitro inhibitory effect on sebum accumulation^82^, besides recognized for their ability to adsorb oiliness and greasiness from the skin^14^. All the aforesaid factors consequently lead to distinctive antiacne activity by the ADA-OPTMS gel.

This was not the case in the Adapalene gel (0.1%); the ADA in the market product was in free form, which, as previously discussed, has poor confinement within the skin layers and hence requires an extended duration of treatment are required to get optimal outcomes. Furthermore, skin exposure to large quantities of ADA may have caused irritation side effects that may have hindered the improvement of the acne condition.

#### Histopathological analysis

##### The first study

**Figure 6:**
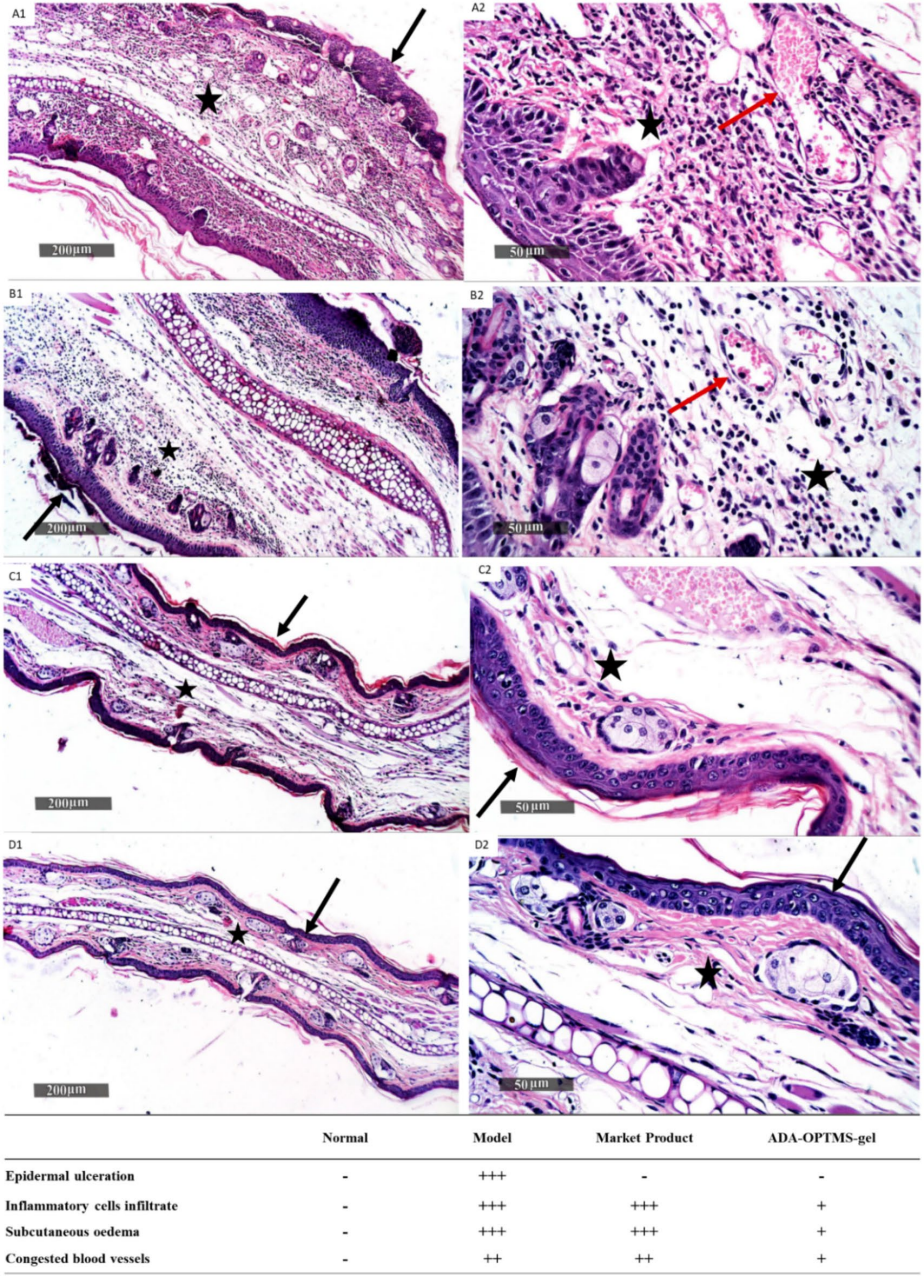
Representative histopathological photomicrographs of skin sections from mice ear group (**A**) model group, (**B)** group receiving Market product Adapalene treatment, (**C**) group receiving ADA-OPTMS-gel treatment and (**D**) Normal mice ear. 1&2: H & E stain micrographs at different magnifications. Black arrow (**→**): Epidermal layer, Star (★): Dermal layer, Red arrow (→): Blood vessels.

The mice of all groups were mercifully sacrificed at the completion of the treatment period, and their auricle sections underwent histopathological examination. The state of the skin architecture, the structural integrity of the epidermal layer, the thickness of the dermal layer, whether there were inflammatory cells, and the condition of the vasculature, as seen in tissue sections in Fig. 6, were used as the basis for the histopathological examination^38^. The histological structures of the various layers are shown in Fig. 6D's photographs of a normal ear. The image shows a continuous dermal layer (star) with minimal inflammatory cells present and normal blood vessels, an intact subcutaneous layer, and a thin outer epidermal layer (arrow) with what appear to be intact, well-ordered keratinocytes, and undamaged subcellular characteristics. This proved that the ear components were free of lesions or other abnormalities. All of the inflammation-related indicators^36^ of severe dermatitis were present in model ear group A (Fig. 6A), together with isolated areas of epidermal necrosis and ulceration that were encased in necrotic tissue deposits (arrow). Additionally, it displayed edema, thickening of the skin layers, and evident significant inflammatory cell infiltrates (star). Dermal and subcutaneous blood vessels are numerous, congested, and dilated, as well as intermittent hemorrhagic patches, as shown by the red arrow. This provided evidence for the evolution of the inflammatory acne paradigm.

Although there was a significant reduction in ear thicknesses of group B mice treated with the market product Adapalene gel (0.1%), the histopathological examination (Fig. 6B) showed severe epidermal thickening with occasional scabs of necrotic tissue depress (arrow) associated with severe edema of the dermal layer as well as severe persistent records of mixed inflammatory cell infiltrates (star). Many congested and dilated dermal and subcutaneous blood vessels were observed (red arrow). These outcomes indicated only a slight enhancement in acne condition, which may be due to skin exposure to the whole dose of ADA at once triggering the associated side effects, including redness, erythema, dryness and desquamation^11^. In addition to the previously discussed results of ADA permeability and retention, where the free ADA from the gel had low confinement in the deeper dermal layers and hair follicles, the inflammatory signs persisted.

Interestingly, group C tissue section examinations (Fig. 6C) showed obviously higher improvement and restoration of normal morphological features among all treated group samples receiving ADA-OPTMS gel. Almost apparent intact epidermal layers (arrow) as well as dermal layers with sporadic few records of inflammatory cell infiltrate (star) and congested blood vessels (red arrow), with minimal edema, are shown. These results combined with the previously established enhanced retention in the epidermal layer and hair follicles and the capability of ADA-OPTMS to hinder sebum accumulation are suggestive of the worthy healing capacity of ADA-OPTMS gel on acne cysts.

##### The second study

Further in-depth inspection using histopathological microimaging for the whole treatment period from day 1 to day 3 using ADA-OPTMS gel was performed (Figure S5). The examination exposed that after 1 day of treatment with ADA-OPTMS gel, a reduction in the progression of acne was portrayed, where the epidermal layers were nearly intact, with occasional records of external minor scabs of necrotic tissue depression. Additionally, the dermal layer was edematous with mild inflammatory cell infiltrates and overall auricular thickening. For day two of treatment, it showed more or less the same records as day one sections. Reaching day three of treatment, the curative effect of the ADA-OPTMS gel was evident.

#### Histochemical analysis

**Figure 7:**
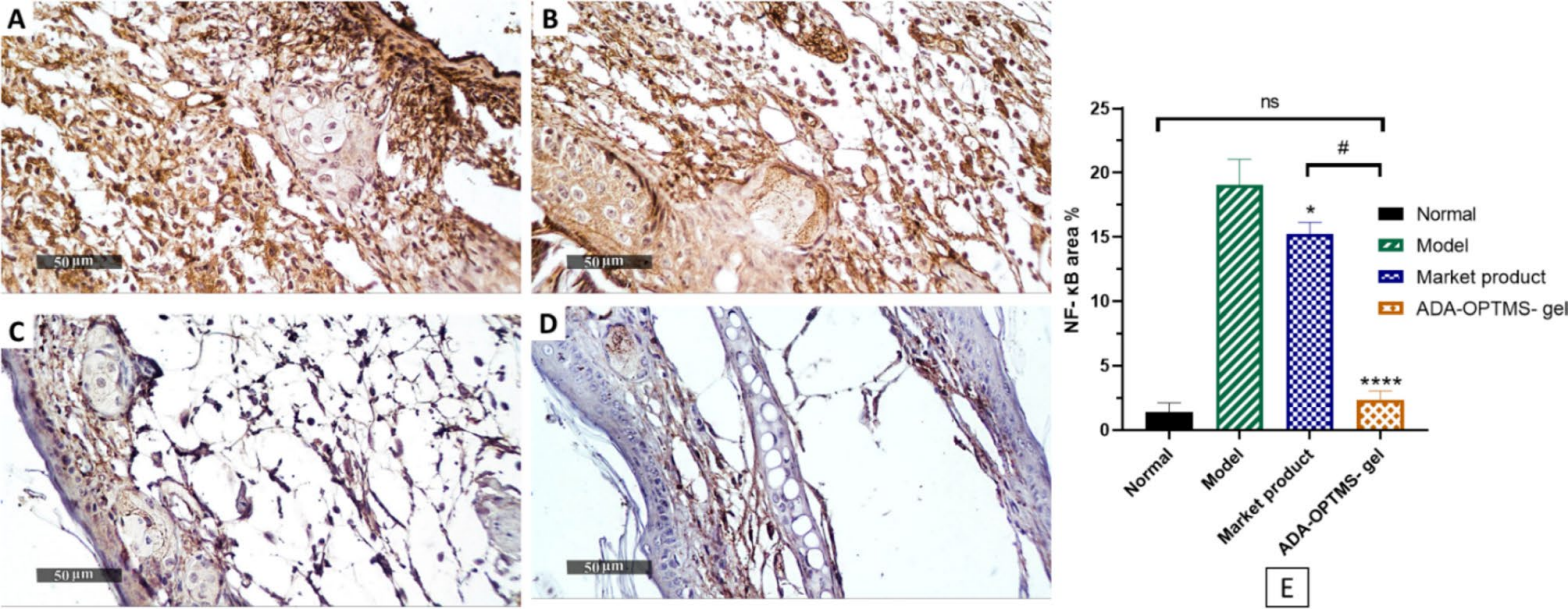
Histochemical analysis of NF-κB levels, (**A**) model group mice ear tissue, (**B**) market product Adapalene group mice ear tissue, (**C**) ADA-OPTMS-gel group mice ear tissue, (**D**) Normal mice ear tissue and E statistical analysis results (Mean ± SD, *n* = 3). The results were compared using one-way ANOVA followed by Tukey’s post hoc test. *p < 0.05; ****p < 0.0001 compared with the model group, ^#^*p* < 0.0001 compared with the market product group and *ns* not significant compared with the normal group.

Confirmatory histochemical analysis of the same tissue sections used in the histopathological examination was conducted on the abundance precent area of nuclear factor kappa B (NF-κB), which is typically triggered in inflammatory acne lesions and aggravates the acne condition^36^. Figure 7 illustrates the findings of the histochemical assay.

The histochemical images of the normal ear exhibited low expression of the inflammatory marker NF-κB (1.4% ± 0.7). Along with, the apparent overexpression of NF-κB in the model group (A) reached 19% ± 2.0, which was significantly higher than that in normal ears. These results were confirmatory of the triumphed induction and progression of acne inflammatory cysts. There was a significant reduction in the NF-κB area percent in group B (market product) mice (15.3% ± 0.9) compared with the model group. These outcomes indicated only a slight enhancement in acne condition as a consequence of the therapeutic effect of ADA. However, the low magnitude of enhancement may be attributable to skin exposure to the total dose of ADA at once triggering the associated side effects, which hinders the resolution of the inflammatory response^11^. Together with the need for prolonged periods of treatment, due to low targeting capabilities into pilosebaceous units. These findings are in agreement with a study performed by Shan et al., 2022, where ADA gel was applied to *P. acnes*-induced microcomedone in mice and achieved a significant reduction in the levels of NF-κB, reaching normal levels after 2 weeks of therapy^42^.

Finally, group C tissue sections showed a practically normal area percent (2.3% ± 0.7) and a highly resilient downregulation of NF-κB. Impressively, there was a very large significant difference between group C and group A and group B in the NF-κB area percentages, but there was no significant difference between group C and the normal ear percentage. This could be explained by the ADA-OPTMS gel having a targeted and controlled release performance^12^, with a trifling skin exposure to the ADA moiety, which achieves the dual effect of protection of the skin from the ADA common irritable side effects and photosensitization effect. Combined with the divulged MS capability to accumulate ADA in the hair follicles in which acne originates, together with the known advantageous characteristics of the microsponges.

These findings proclaim the superlative effectiveness of ADA-OPTMS gel over the market product Adapalene in the remedy of acne disease with subsided inflammatory side effects within only 3 days of treatment.

### Clinical study

Retinoid derivatives have been considered highly advantageous in the treatment of mild to moderate acne diseases and are often prescribed by physicians^83^. They are, however, frequently accompanied by local irritation side effects. After the promising in vivo results of the ADA-OPTMS gel in the treatment of acne with lower irritation potential, henceforward clinical evaluation was encouraged. Thus, the purpose of this split-face preliminary comparative clinical study was to assess the efficacy and tolerability of the proposed DAP, ADA-OPTMS gel (0.1%ADA), in comparison with MAP, the well-known market gel Adapalene (0.1%), in combating acne disease. Table 4 shows the baseline characteristics of the population. A total of ten patients with mild to moderate facial acne vulgaris, 7 females and 3 males, with a mean age of 22.1 ± 4.6 years and median duration of the disease of 7 months, were enrolled. DAP was applied to the right side, while MAP was applied to the left side once daily for twelve consecutive weeks.

**Table 4:** The demographic characteristics of the patients enrolled in the clinical study (DAP vs MAP).

Characteristics	Descriptive statistics N = 10
Age (years)	Range: 16–30
	Mean ± SD: 22.1 ± 4.6
Sex (number, %)	Male: 3 (30%)
	Female: 7 (70%)
Duration of disease (months)	Median: 7
	IQR: 3.8–9

#### Primary clinical assessment

**Figure 8:**
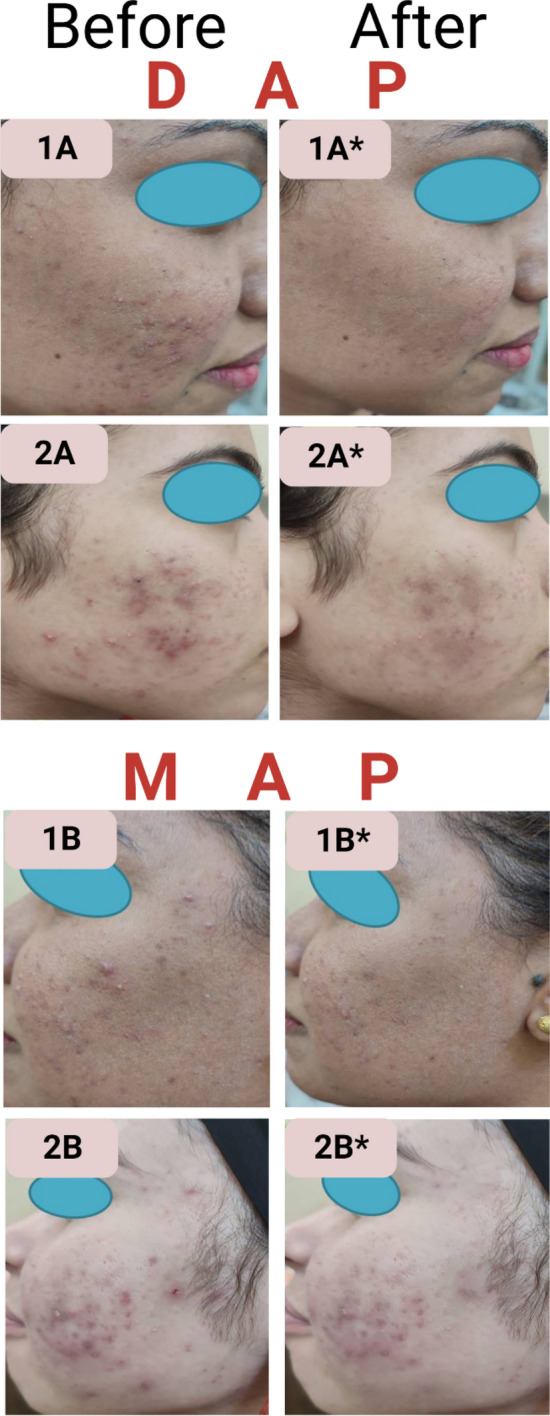
Representative images before and after DAP versus MAP treatment. (**A**) Patient receiving DAP before treatment, (A*) Patient receiving DAP after treatment, (**B**) patient receiving MAP before treatment, and (B*) patient receiving MAP after treatment (1 and 2 patients’ numbers). Photos publication is approved by the patients.

The outcomes of the study are displayed in Table 5 and Fig. 8. At baseline, concerning the number of total, noninflammatory and inflammatory lesions, there was no significant difference between the two sides of the face. This reflected a similar severity of acne conditions at the start of the study.

**Table 5:** Assessment of patients’ acne lesions count pre and post treatment using DAP versus MAP.

Characteristics	DAP	MAP	*p* value
	Median IQR	Median IQR	
Non-inflammatory before	5	4.5	0.7581
	(3.8–10.5)	(2.8–10.8)	
Non-inflammatory after	0	2	**0.03**
	(0–0.5)	(0–5)	
Reduction %	100	60.8	**0.007**
	(95.8–100)	(50–100)	
*p* value (before vs after)	**0.002**	**0.004**	
Inflammatory before	6.5	6	0.896
	(2.8–13)	(3.3–9.8)	
Inflammatory after	2	3	0.347
	(0–3)	(0.8–4.5)	
Red. %	75.7	61.3	0.133
	(65.7–100)	(50–81.3)	
*p* value (before vs after)	**0.005**	**0.005**	
Total before	14	15	1
	(9–19.5)	(6.5–19.3)	
Total after	3	7.5	**0.0497**
	(0–3.3)	(1.5–8.3)	
Reduction %	82.9	59.7	**0.021**
	(76.9–100)	(50–78.6)	
*p* value (before vs after)	**0.002**	**0.002**	

Mann Whitney test for non-parametric quantitative data between the two groups.

Wilcoxon Signed rank test for non-parametric quantitative data between the two times within each group.

Significant values are in bold

*IQR* interquartile range, N = 10.

Significance level at *p *value ≤ 0.05.

For the posttreatment evaluation, both DAP and MAP demonstrated improvement in the acne condition over the treatment period. However, a gradual reduction in the number of lesions was observed on both sides during the follow-up visits reaching the twelfth week of treatment. There was a highly significant difference between the total number of lesions prior to and after treatment on both sides of the face. On the other hand, the total and the precent reduction of the lesions on the DAP face side was significantly higher than that on the MAP face side. This result showed an improvement trend in the overall treatment when DAP was applied.

Moving on to the noninflammatory lesions. Even though both treatments presented a significant decrease in the number of noninflammatory lesions before and after treatment, DAP demonstrated a significantly higher reduction in the number and percent reduction of noninflammatory lesions than MAP. Almost 80% of the subjects reached clear grades when using DAP compared with only 30% when using MAP. This is probably a result of the improved delivery of ADA to the epidermal layer and the pilosebaceous units. As well as the improved hydration of skin via the occlusive effect of the MS system in the ADA-OPTMS gel plays a role in improved targeted topical delivery through skin barriers. The ability of microsponges to absorb oil up to 6 times its weight^70^ aids in the sebum control process. This could help understand the better efficacy of the ADA-OPTMS gel (0.1% ADA) compared to the marketed gel Adapalene (0.1%) despite using the same concentration of the same active ingredient.

Conversely, the inflammatory lesions, despite the decreasing trend in the number and percent reduction of lesions on the face side using DAP, were higher than those of MAP, hitherto; however, the difference between them was found to be insignificant. This may be attributed to the small sample size in the study^84^. Even though in the present study, ADA, along with the known RAR binding mechanism of action, was found to attain antimicrobial activity that was enhanced when encapsulated into MS, the MIC was relatively high, and thus, a longer duration of treatment is probably required to achieve a significant decrement trend. Nevertheless, the observed clinical improvement in terms of lesion count, size and visibility was in favor of the DAP-treated side.

Comparable findings were discovered in a 12-week randomized clinical study conducted by Najafi-Taher et al. in 2022. An adapalene gel-containing tea tree oil nanoemulsion (TTO NE + ADA gel) was contrasted with commercially available ADA gel. When compared to the ADA marketed gel group, the results showed that the group treated with TTO NE + ADA gel had significantly decreased total, inflammatory, and noninflammatory acne lesions^44^. This was attributed by the authors to the anti-inflammatory properties of TTO coupled with ADA in a nanosized carrier, which enhanced therapeutic impact by improving active drug distribution to pilosebaceous units.

#### Tolerability assessment

The irritation assessment revealed that DAP was better tolerated than MAP. Erythema, pruritus, scaling, and burning sensations were frequently present with high severity on the MAP left face side, and all the patients reported that irritation side effects varied from mild (20%), moderate (60%) and severe (20%). On the other hand, 70% of the patients reported no irritation side effects on the right side treated with DAP, and only 30% reported mild irritations. DAP was found to be significantly more tolerable than MAP (*p* value = 0.001). This could be explicated by the ADA-OPTMS gel establishing targeted controlled release behavior with pointed minimization of skin exposure to the total dose of ADA, accomplishing protection of the skin from the reputed ADA side effects. Regarding safety measures, there were no reported severe adverse events that required discontinuation in either treatment.

Other research with similar findings concluded that tolerance is enhanced when ADA is encapsulated in carrier systems^11^. For instance, Rao et al.^85^ conducted a randomized, assessor-blind, multicentric comparative, postmarketing phase IV trial where microsphere adapalene gel and standard adapalene gel were juxtaposed for 12 weeks of treatment. The study's findings showed that the usage of the microsphere ADA gel significantly reduced dryness and erythema and that using the traditional adapalene gel resulted in a number of patients stopping their therapy because of severe irritant adverse effects^85^. Another study found that tretinoin in microsponges produced larger decreases in both inflammatory and noninflammatory lesions^86^.

Consequently, it could be concluded that the ADA-OPTMS gel is highly auspicious in the amelioration of patient compliance due to its heightened efficacy and tolerability.

#### Secondary clinical assessment

The physician’s global assessment (PGA), as shown in Table 6a, revealed that the global improvement of acne severity for the overall treatment on both sides was efficient. A significant difference between pre- and post-evaluation of treatment with DAP and MAP was perceived. Concerning the face side treated with DAP, the physicians had the impression of a better resolution of the acne condition, with 80% clear and only 20% of the patients demonstrating remaining comedones. The MAP face side showed 0% complete clearance, 60% almost clear clearance and 40% mild clearance of the comedones. According to the PGA, the DAP treatment was significantly better than the MAP treatment.

**Table 6:** (a) Physician assessment score before and after treatment using DAP versus MAP, (b) patient assessment score before and after treatment using DAP versus MAP.

	DAP N (%)	MAP N (%)	*p *value
**(a) Physician assessment score**			
Before			
Clear	0	0	0.526
Almost clear	0	0	
Mild	1 (10%)	1 (10%)	
Moderate	6 (60%)	8 (80%)	
Severe	3 (30%)	1 (10%)	
After			
Clear	8 (80%)	0	**0.001**
Almost clear	2 (20%)	6 (60%)	
Mild	0	4 (40%)	
Moderate	0	0	
Severe	0	0	
*p* value (before vs after)	< 0.001	0.001	
**(b) Patient satisfaction score**			
Dissatisfied	0	0	**0.02**
Slightly satisfied	0	3 (30%)	
Moderately satisfied	3 (30%)	5 (50%)	
Full satisfied	7 (70%)	2 (20%)	

Significance level at *p *value ≤ 0.05.

Synchronously, the patient’s global assessment (PaGA), as presented in Table 6b, revealed that the treatment with DAP was much more satisfactory by the patients than MAP. The PaGA demonstrated that 70% of the patients were fully satisfied and only 30% were moderately satisfied with DAP treatment. On the other hand, only 20% were fully satisfied, 50% were moderately satisfied and 30% were slightly satisfied with the MAP treatment. Overall, ADA-OPTMS gel was particularly well tolerated and effective in this patient population of people with mild to moderate acne.

Both the commercially available adapalene and the ADA-OPTMS gel significantly improved the acne condition in the current clinical research over the course of the treatment period (12 weeks). The two therapies had a similar impact on inflammatory acne lesions. However, in reducing noninflammatory comedonal lesions, the ADA-OPTMS gel was identifiable from commercial adapalene, demonstrating its excellent comedolytic action. Additionally, it was clearly proven that this novel formulation had a remarkably minimal propensity for discomfort. Furthermore, the ADA-OPTMS gel was preferred by both the physicians and the patients in the treatment of acne disease, encouraging futuristic technology transfer.

## Conclusion

The present study results revealed that the Eudragit RS100 percent in polymer mixture, organic phase volume, and drug (ADA) to polymer mixture percent indeed significantly influenced the dependent parameters, P. Y%, E.E.%, P.S., and Q24h, of the prepared adapalene-loaded microsponges. Additionally, BBD was successful in optimizing the preparation conditions for ADA-OPTMS, which were found to be 43.1% of EUD, 15 ml of DCM, and 70%:30% of ADA to polymer mixture percent. The optimized formula exhibited a homogeneous, spongy, spherical structure and attained biphasic controlled release. The incorporation of ADA-OPTMS in the hydrogel base was practical and had amiable characteristics. The following skin deposition studies both qualitatively and quantitatively demonstrated the targeted pattern of delivery of ADA from the MS with the highest confidence within the pilosebaceous units, the acne active site. Further in vivo investigations, together with histopathological and histochemical analysis, confirmed the superiority of the ADA-OPTMS gel compared with the market Adapalene gel in the treatment of acne conditions and subsiding the associated inflammatory conditions. Last, the previous results of efficacy were inveterate by the clinical study, along with the significant enhancement of tolerability and thus patient compliance. Accordingly, it could be concluded that the MS are an efficient carrier system for ADA and that the ADA-OPTMS-gel is competent in the targeted treatment of acne disease with plummeted side effects and systemic absorption, enhancing patient compliance compared to previously developed approaches for the delivery of adapalene. Thus, futuristic commercialization is envisioned.

### Supplementary Information

Supplementary Information.

## Data Availability

The datasets generated or analyzed during the current study are available from the corresponding author upon reasonable request.
