# Supplementary material for: Resolving acne with optimized adapalene microspongeal gel, in vivo and clinical evaluations
Source: Sci Rep. 2024 Jan 16;14:1359. doi: 10.1038/s41598-024-51392-1 (PMC10791745; doi:10.1038/s41598-024-51392-1)
Supplement: Supplementary file 1 — Supplementary Information. [file 41598_2024_51392_MOESM1_ESM.docx]

**Supplementary Information**

**Resolving Acne with Optimized Adapalene Microspongeal Gel, In Vivo and Clinical Evaluations**

**Rania M. Yehia^1^, Mahmoud H. Teaima^2^,** **Maha H. Ragaie^3^, Mohamed M. Elmazar^4^, Dalia A. Attia^1^ and Mohamed A. El-Nabarawi^2^**

^1^Department of Pharmaceutics and Pharmaceutical technology, Faculty of Pharmacy, The British University in Egypt (BUE), Cairo, Egypt.

^2^Department of Pharmaceutics and Industrial Pharmacy, Faculty of Pharmacy, Cairo University, Cairo, Egypt.

^3^Department of Dermatology, STD’s and Andrology, Faculty of Medicine, Minia University, Al Minya, Egypt.

^4^Department of Pharmacology and Toxicology, Faculty of Pharmacy, The British University in Egypt (BUE), Cairo, Egypt.

**Table (S1): The generated model values and equations for each parameter**

| **Model** | **P.Y. % (Y1)** | **E.E. % (Y2)** | **P.S. (Y3)** | **Q 24h (Y4)** |
| --- | --- | --- | --- | --- |
| **Lack of fit** | 5.8 | 0.31 | 0.38 | 8.2 |
| **R^2^** | 0.966 | 0.936 | 0.942 | 0.961 |
| **Adequate precision** | 13.75 | 7.98 | 9.22 | 12.22 |
| **Equation** | *= +91.08 – 5.83 A +0.800 B – 0.5938 C +3.96 AB – 4.94 AC + 2.44 BC – 5.94 A^2^ +0.4677 B^2^ +4.21 C^2^* | *=+77.13 – 0.5019 A – 2.46 B – 3.86 C + 2.33 AB – 8.32 AC – 2.37 BC + 1.45A^2^ + 5.67 ^B2^ +10.91 C^2^* | *=+21.33 – 6.96 A – 5.09 B – 0.500 C – 2.25 AB – 2.92 AC + 1.33 BC + 7.88 A^2^ + 0.6833 B^2^ +2.06 C^2^* | *=+62.77 + 1.88 A – 8.53 B + 4.67 C + 5.31 AB + 5.82 AC + 18.08 BC – 16.44 A^2^ – 2.09 B^2^ – 8.56 C^2^* |

**Table (S2):** **The predicted, observed, residual values and bias percent of the responses for the optimized formula**

| **Responses** | **Predicted value** | **Observed**  **value** | **Residuals** | **Bias %** |
| --- | --- | --- | --- | --- |
| Production Yield % | 98.55 | 98.3 | 0.250 | 0.2543 |
| Entrapment efficiency % | 96.9 | 97.3 | -0.400 | -0.4111 |
| Particle size | 31.385 | 31.8 | -0.415 | -1.3050 |
| Cumulative drug release after 24 h % | 75.026 | 75.1 | -0.074 | -0.0985 |

**Table (S3): The validation parameters of the UPLC analysis method**

| **Parameter** | **Value** |
| --- | --- |
| Regression coefficient (r2) | 0.9984 |
| Precision | 5.3% |
| Accuracy | 98.6% |
| Limit of detection | 0.0314 µg/ml |
| Limit of quantification | 0.0952 µg/ml |
| Ruggedness | 1.766% |

**Table (S4): The distribution of pixels in between skin layers from the prepared Dil-F loaded OPTMS gel and Dil-F loaded plain gel**

| **Formulae** | **Skin layers pixels distribution (pixel/μm^2^)** | | |
| --- | --- | --- | --- |
|  | Stratum corneum | Epidermis | Dermis |
| Dil-F loaded OPTMS gel | 104.93 ± 9.62 | 190.56 ± 13.83* | 132.53 ± 13.49* |
| Dil-F loaded plain gel | 135.2 ± 6.35* | 105.07 ± 8.54 | - 1. 8.06 |

^*Significantly higher value^ *^p-value^* ^<0.05^


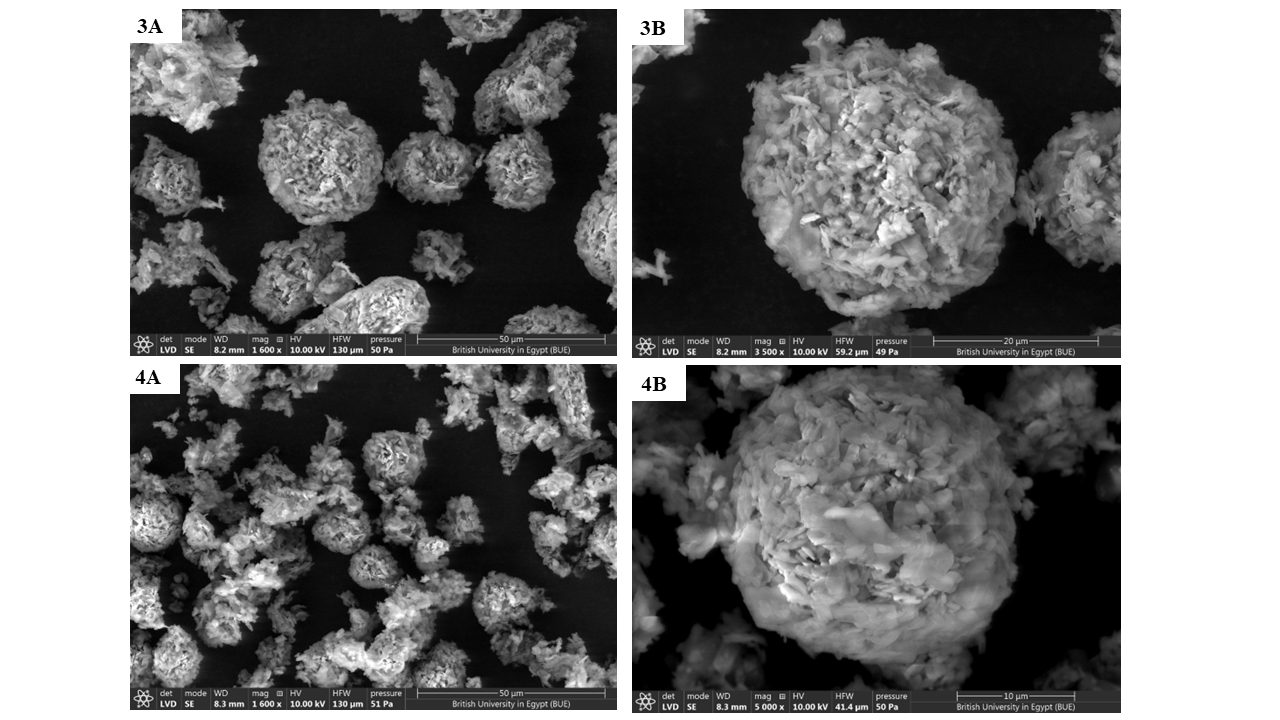
**
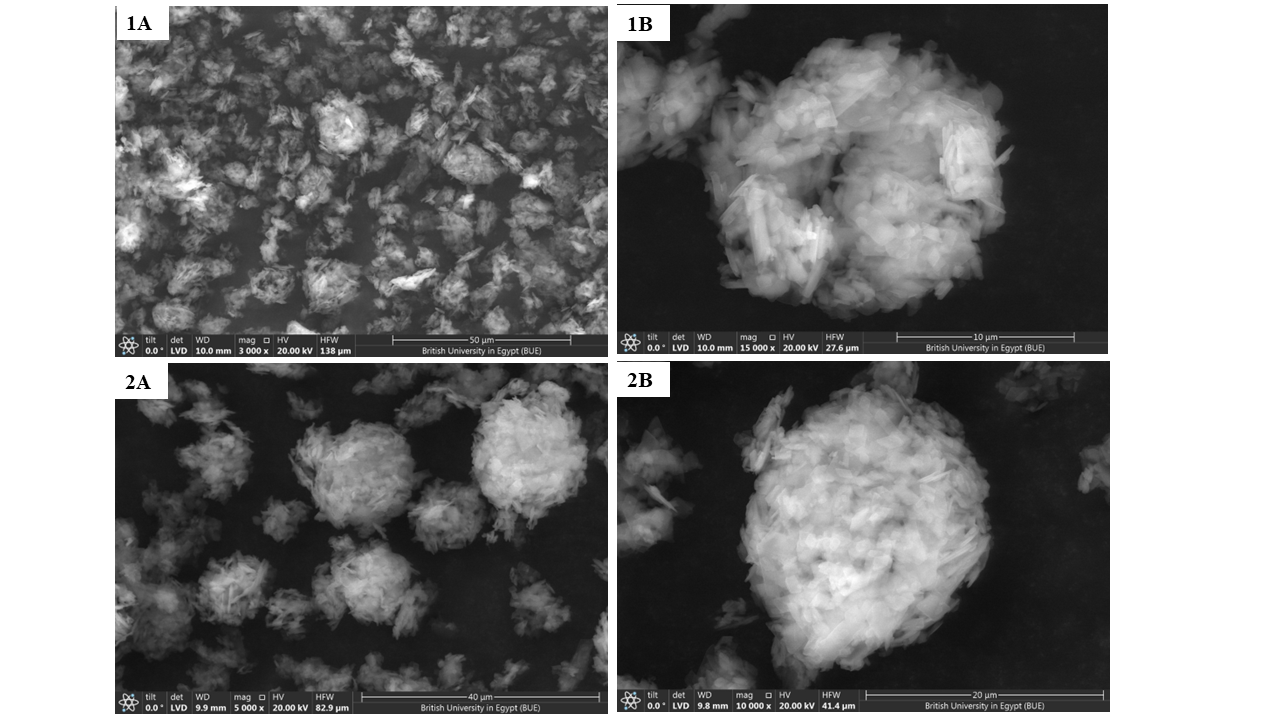
**


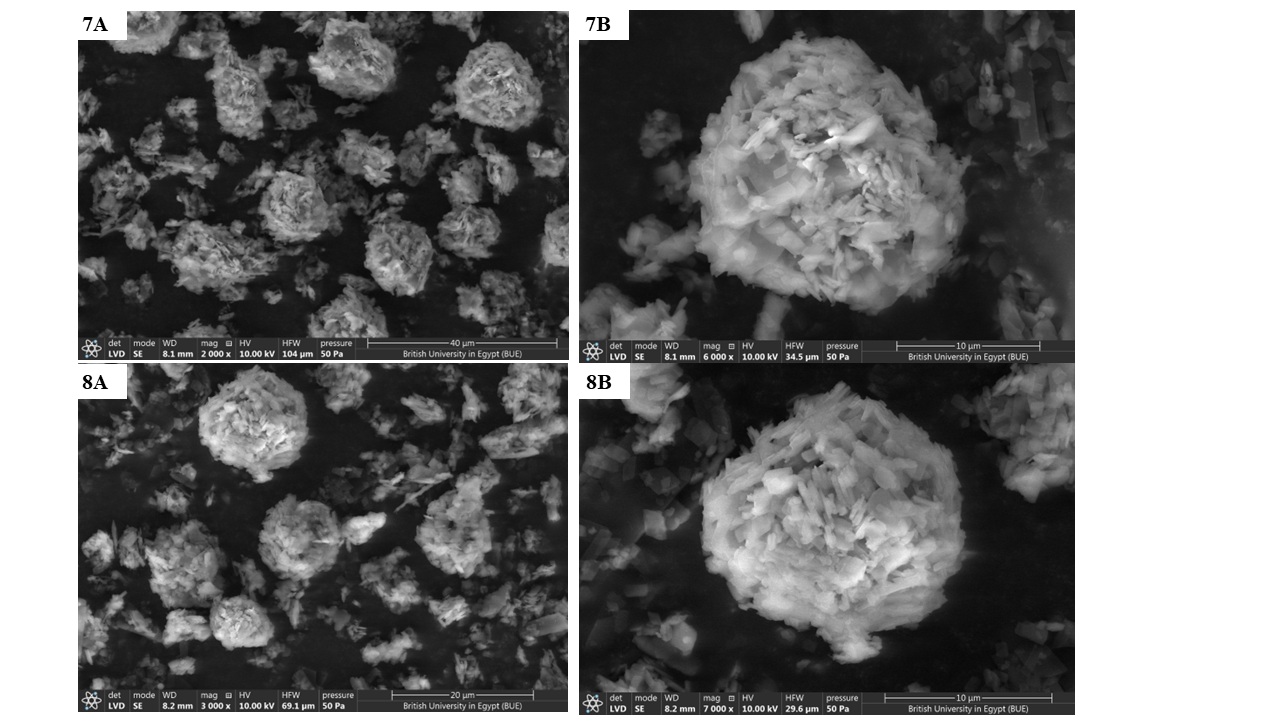
**
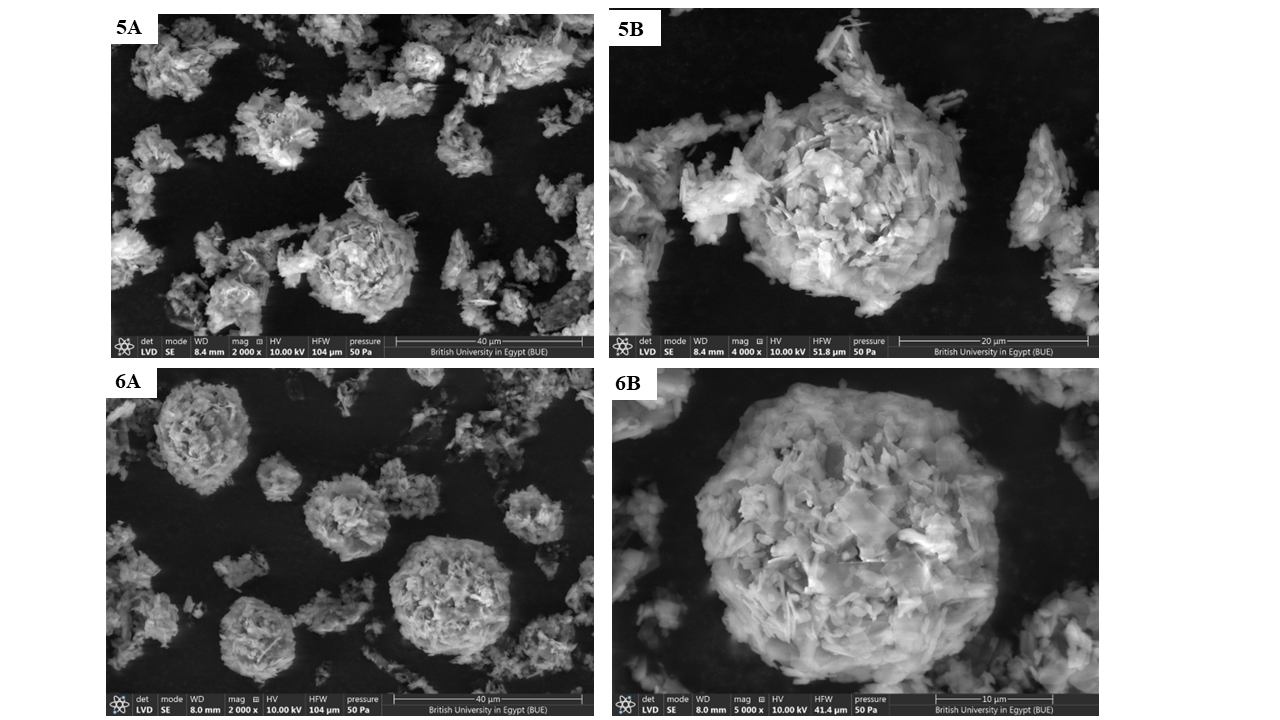
**


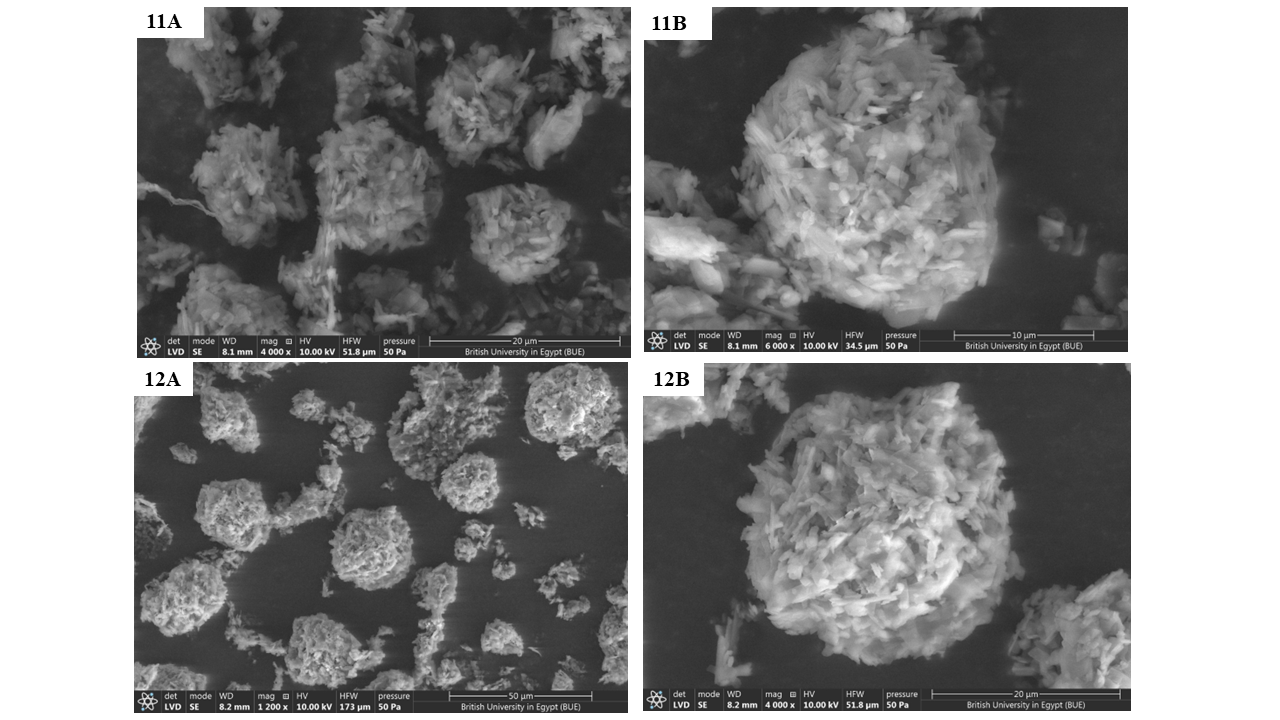

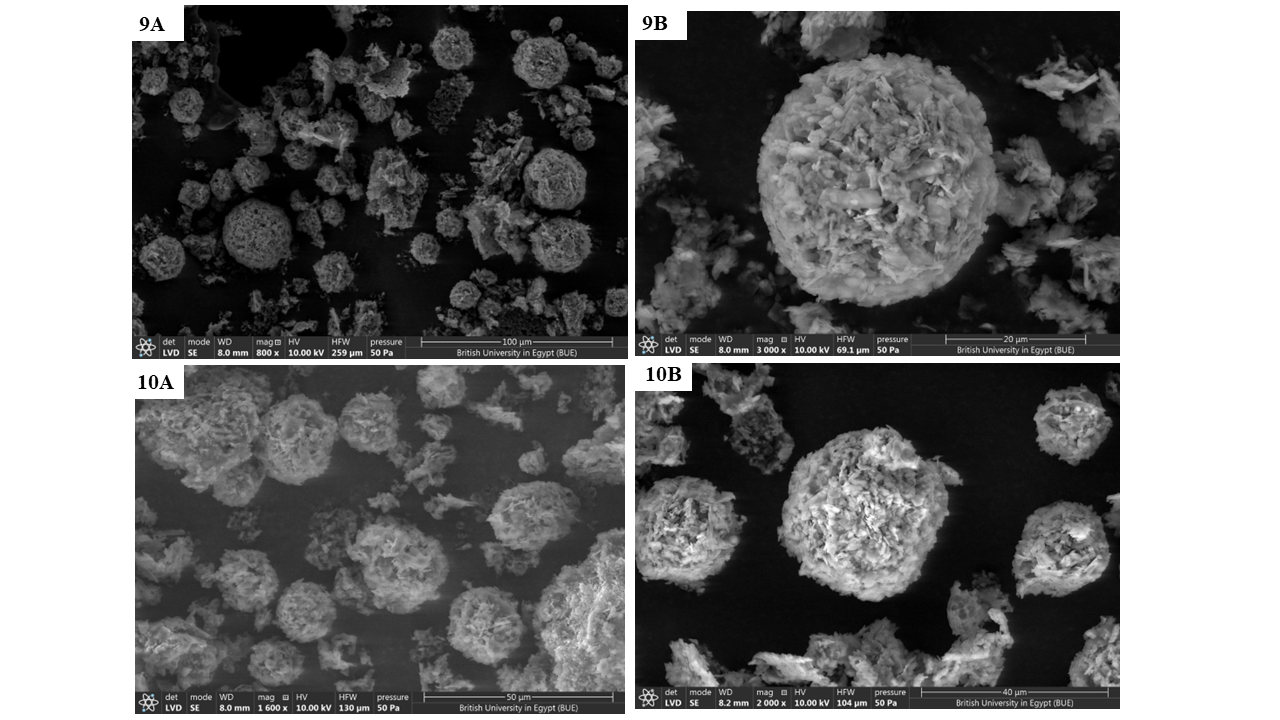


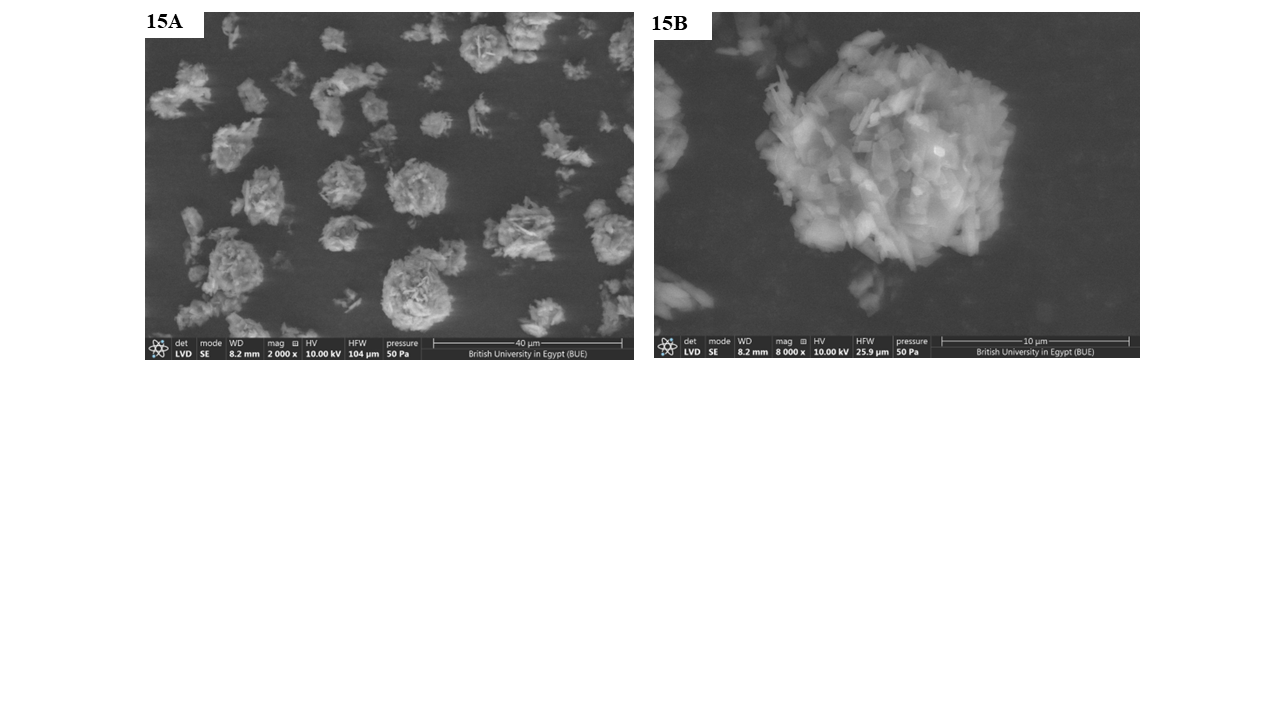

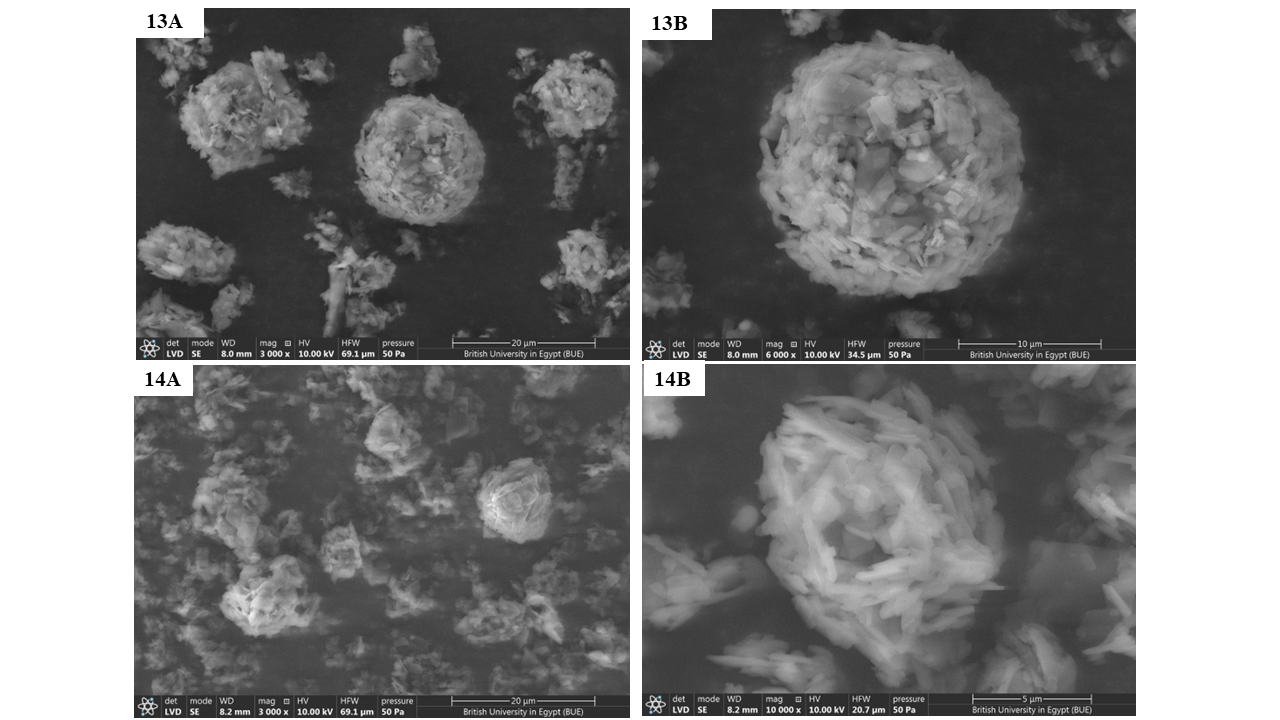


**Figure (S1): The SEM images of F1 to F15, where A: showing images of the field and B: showing images of a single microsponge particle.**

**Figure (S2a): *In vitro* release profile of ADA-MS formulae prepared using Eudragit RS 100 (F1, F4, F5 and F14).**

**Figure (S2b): *In vitro* release profile of ADA-MS formulae prepared using Ethyl Cellulose (F3, F6, F9 and F12).**

**Figure (S2c): *In vitro* release profile of ADA-MS formulae prepared using Eudragit RS 100 and Ethyl Cellulose 1:1 mixture (F2, F7, F8, F10, F11, F13 and F15).**


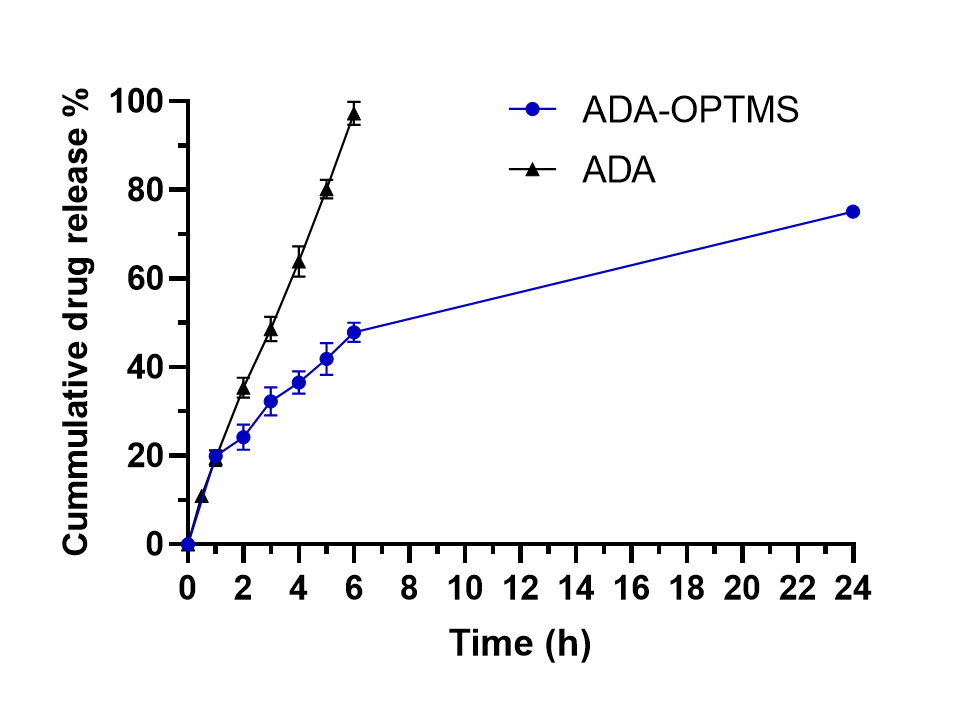


**Figure (S3): The in vitro release profile of the optimized adapalene-loaded microsponges formula and pure adapalene.**

**
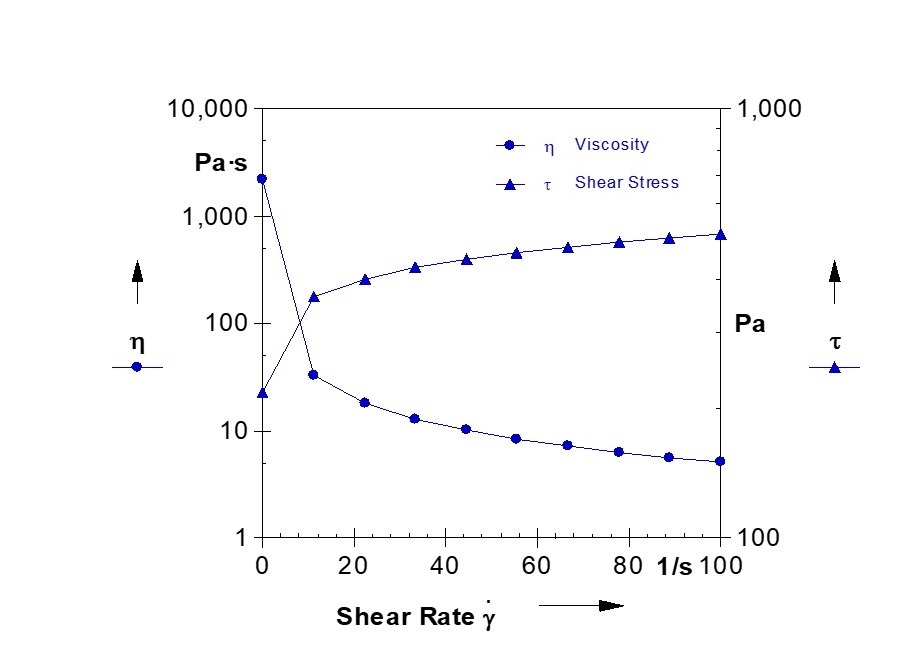
**

**Figure (S4): Rheogram of the prepared ADA-OPTMS gel**


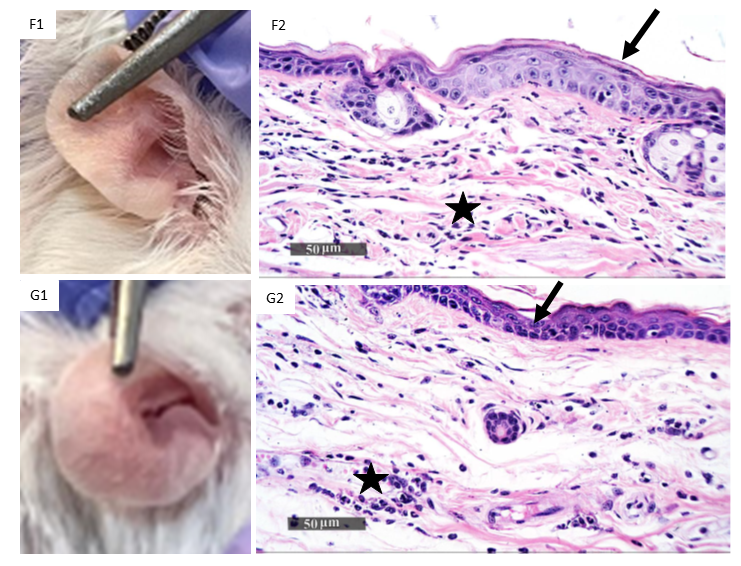


**Figure (S5). Representative daily changes of mice ears of groups (n=3) receiving ADA-OPTMS-gel.** F: Day 1, G: Day 2. 1: Digital photo-macrographs and 2: H & E stain micrographs. Black arrows indicate epidermal layer, the stars show the dermal layer tissue with inflammatory cells infiltrates.
